# A novel family of proline/serine-rich proteins, which are phospho-targets of stress-related mitogen-activated protein kinases, differentially regulates growth and pathogen defense in *Arabidopsis thaliana*

**DOI:** 10.1007/s11103-017-0641-5

**Published:** 2017-07-28

**Authors:** Mieder Anthony Thomas Palm-Forster, Lennart Eschen-Lippold, Joachim Uhrig, Dierk Scheel, Justin Lee

**Affiliations:** 1 Leibniz Institute of Plant Biochemistry, Weinberg 3, 06120 Halle/saale, Germany; 2 Eurofins Lancaster Laboratories, 2425 New Holland Pike, Lancaster, PA 17605 USA; 3 Department of Plant Molecular Biology and Physiology, Georg August University of Goettingen, Julia-Lermontowa-Weg 3, 37077 Goettingen, Germany

**Keywords:** MAPK, PAMPs, Pathogen resistance, Phosphorylation, Plant development, Oxidative stress

## Abstract

The molecular actions of mitogen-activated protein kinases (MAPKs) are ultimately accomplished by the substrate proteins where phosphorylation affects their molecular properties and function(s), but knowledge regarding plant MAPK substrates is currently still fragmentary. Here, we uncovered a previously uncharacterized protein family consisting of three proline/serine-rich proteins (PRPs) that are substrates of stress-related MAPKs. We demonstrated the importance of a MAPK docking domain necessary for protein–protein interaction with MAPKs and consequently also for phosphorylation. The main phosphorylated site was mapped to a residue conserved between all three proteins, which when mutated to a non-phosphorylatable form, differentially affected their protein stability. Together with their distinct gene expression patterns, this differential accumulation of the three proteins upon phosphorylation probably contributes to their distinct function(s). Transgenic over-expression of PRP, the founding member, led to plants with enhanced resistance to *Pseudomonas syringae* pv. *tomato* DC3000. Older plants of the over-expressing lines have curly leaves and were generally smaller in stature. This growth phenotype was lost in plants expressing the phosphosite variant, suggesting a phosphorylation-dependent effect. Thus, this novel family of PRPs may be involved in MAPK regulation of plant development and / or pathogen resistance responses. As datamining associates *PRP* expression profiles with hypoxia or oxidative stress and PRP-overexpressing plants have elevated levels of reactive oxygen species, PRP may connect MAPK and oxidative stress signaling.

**Electronic supplementary material:**

The online version of this article (doi:10.1007/s11103-017-0641-5) contains supplementary material, which is available to authorized users.

## Introduction

Plants employ an elaborate signaling network to establish the necessary defense responses to fend off invading pathogens. Recognition of highly conserved microbial structures (so-called microbe/pathogen-associated molecular patterns, MAMPs / PAMPs), by membrane-resident pattern recognition receptors (PRRs), trigger a vast array of responses including ion fluxes across the plasma membrane, an oxidative burst, mitogen-activated protein kinase (MAPK) signaling cascades, changes in phytohormone levels, defense-related gene expression and synthesis of secondary antimicrobial metabolites; all of which lead to PAMP-triggered immunity (PTI); (Couto and Zipfel 2016). The most intensively studied PAMPs are flg22, a 22 amino acid motif of bacterial flagellin, and elf18, an 18 amino acid motif found in the bacterial elongation factor EF-Tu (Boller and Felix 2009).

MAPK cascades are central regulatory modules involved in diverse (stress) adaptation processes, as well as developmental aspects. They are organized in a hierarchical order, typically consisting of three kinases (MAP3K, MAP2K, MAPK) that activate their respective downstream kinases (Suarez Rodriguez et al. 2010). Activated MAPKs, in turn, phosphorylate a broad range of substrate proteins to modify their stability, activity or localization. Further evidence of the multi-level cellular reprogramming in response to MAPK signaling have been identified through the observation of complex changes in the phospho-proteome of transgenic plants with inducible MAPK activation (Hoehenwarter et al. 2013; Lassowskat et al. 2014; Lee et al. 2015).

Two branches of MAPK signaling were described in *Arabidopsis thaliana* to be activated upon PAMP recognition, one involving MEKK1-MKK1/2-MPK4 (Ichimura et al. 2006), the other including a still unidentified MAP3K, MKK4/5 and MPK3/6 (Asai et al. 2002). Apart from MPK3/4/6, another MAPK, MPK11, is known to be activated upon PAMP treatment (Bethke et al. 2012; Eschen-Lippold et al. 2012). Knowledge of plant MAPK substrate proteins is still scarce. In several studies, large scale identification of putative substrates has been performed (Benschop et al. 2007; Feilner et al. 2005; Hoehenwarter et al. 2013; Lassowskat et al. 2014; Popescu et al. 2009), but functional data is limited. A few examples for defense-related substrates analyzed in detail are PAT1, an mRNA decay factor (Roux et al. 2015), ERF104, an ethylene response factor (Bethke et al. 2009), ACS2/6, two proteins involved in ethylene biosynthesis (Han et al. 2010; Liu and Zhang 2004), VIP1, a bZIP transcription factor (Djamei et al. 2007), the VQ-motif-containing proteins MKS1 and MVQ1 (Andreasson et al. 2005; Pecher et al. 2014), the WRKY transcription factors WRKY33 and WRKY46 (Mao et al. 2011; Sheikh et al. 2016) and TZF9, a tandem zinc finger protein involved in post-transcriptional regulation (Maldonado-Bonilla et al. 2014).

MAPK signaling is also of great importance for plant development, as is illustrated by the growth defects of several MAPK pathway mutants. This includes *mekk1* and *mpk4*, the double mutants *mkk1*/*2* and *mpk3*/*6*, as well as the triple mutant *anp1*/*2*/*3*; all are severely dwarfed and/or show seedling lethality (Gao et al. 2008; Ichimura et al. 2006; Krysan et al. 2002; Nakagami et al. 2006; Petersen et al. 2000; Qiu et al. 2008; Wang et al. 2007). Stomatal development and patterning is controlled by the MAPK cascade YODA-MKK4/5-MPK3/6 (Wang et al. 2007) and cytokinesis requires the MAPK cascade ANP1/2/3-MKK6(ANQ)-MPK4 (Takahashi et al. 2010). The involvement of the same MAPKs in different regulatory contexts, i.e. plant development and adaptive defense processes, implies that MAPKs exist in multifunctional modules. The question of signal fidelity maintenance is a longstanding research field for MAPK-related studies, where the repertoire of pathway-specific MAPK interactors and substrates is undoubtedly a key part of the answer.

In our efforts to identify new MAPK interactors, we conducted Yeast-2-Hybrid screening (Y2H) with MPK3, MPK4, MPK6 and MPK11 as baits. We isolated a protein, which belongs to a class of uncharacterized proteins with three members in *Arabidopsis thaliana*. Here, we present protein–protein interaction studies and the functional characterization of these proteins with respect to pathogen defense regulation and plant development.

## Results

### Members from a family of proline/serine-rich proteins (PRPs) interact with MAPKs

**Fig. 1:**
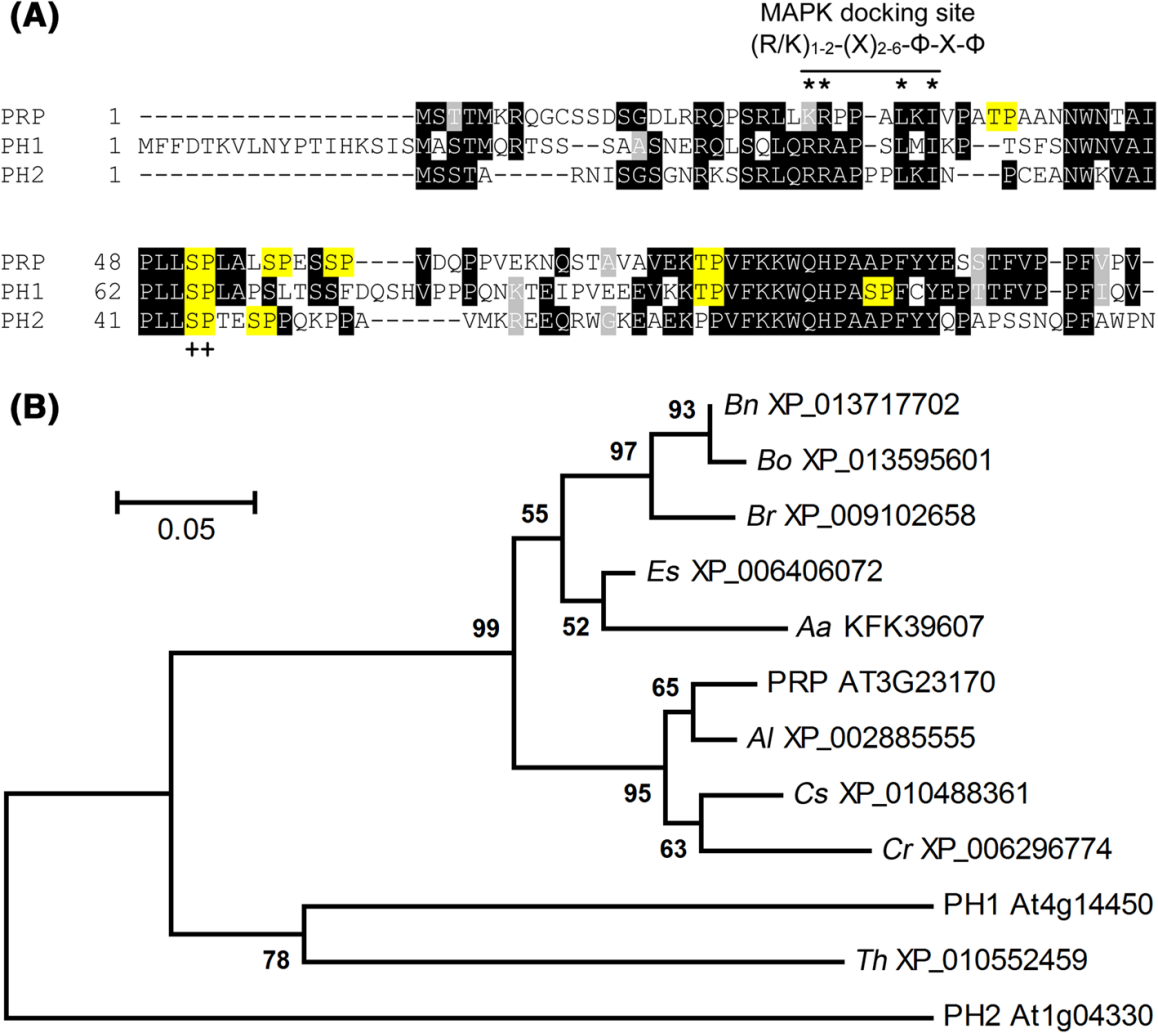
Sequence and phylogenetic analyses of PRP, PH1 and PH2. **a** *Box shade* representation of a multiple sequence alignment of PRP, PH1 and PH2. Putative MAPK phosphorylation sites ([S/T]P) are highlighted in *yellow; ++* indicates a putative MAPK phosphorylation site conserved in all three proteins. Also indicated is a conserved MAPK docking site (R/K)_1−2_-(X)_2−6_-Φ-X-Φ, where *Φ* represents a hydrophobic residue, *X* stands for any residue (PRP: K26, R27, L31, I33; PH1: R42, R43, L47, I49; PH2: R21, R22, L27, I29). Molecular weights: PRP 11.7 kDa, PH1 14.1 kDa, PH2 11.3 kDa; proline content: PRP 15.9%, PH1 12.8%, PH2 18.0%. **b** Phylogenetic analysis of PRP, PH1, PH2 and close homologs from other species. GenBank identifiers are given next to the species abbreviations (*Bn Brassica napus* cv. ZS11, *Bo Brassica oleracea* var. *oleracea, Br Brassica rapa* cv. Chiifu-401-42, *Es Eutrema salsugineum, Aa Arabis alpina* cv. Pajares, *Al Arabidopsis lyrata* subsp. *lyrata, Cs Camelina sativa* cv. DH55, *Cr Capsella rubella* cv. Monte Gargano, *Th Tarenaya hassleriana*). Details are given in the “Materials and Methods”

Yeast two-Hybrid (Y2H) screening identified a protein of unknown function (encoded by the gene locus At3g23170) as an interactor of defense-related MAPKs. This was recovered in 4 and 14 independent clones isolated with MPK11 or MPK6 as Y2H baits, respectively. The predicted open reading frame (ORF) encodes a 107-amino-acid protein with high content of proline (15.9%) and serine (11.2%) and we therefore tentatively named it proline/serine-rich protein (PRP). There are two homologs in the* Arabidopsis* genome, At4g14450 and At1g04330, which we hereafter refer to as PRP Homolog 1 and 2 (PH1, PH2), respectively (Fig. 1a). Database sequence searches revealed putative orthologues in numerous dicotyledonous plants (Fig. 1b). Notably, no homologs were found in monocots or primitive plants (Fig. S1), suggesting *PRP-*like genes presumably evolved after the monocot-dicot divergence.

**Fig. 2:**
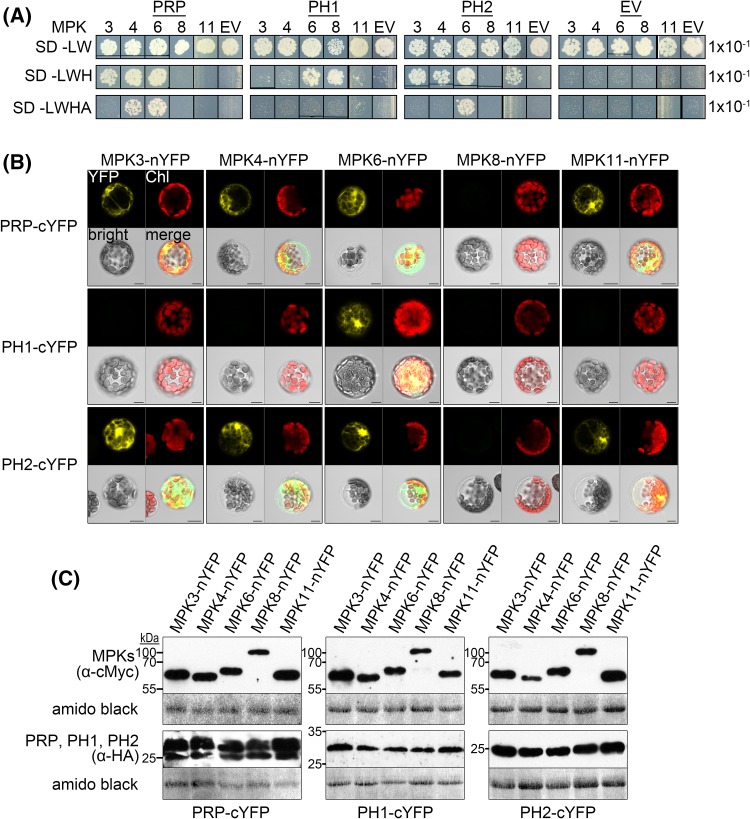
PRP, PH1 and PH2 interact with MAPKs in vivo. **a** Yeast two-Hybrid (Y2H) interaction analyses of PRP, PH1 and PH2 proteins with *Arabidopsis* MAPKs. Positive interactions are indicated by yeast growth on selective synthetic drop-out media (SD-Leu/-Trp/-His or SD-Leu/-Trp/-His/-Ade); *EV* empty vector control (pDEST32). **b** Bimolecular fluorescence complementation (BiFC) assay in* Arabidopsis* protoplasts transiently expressing fusions of PRP, PH1 or PH2 with a C-terminal Yellow Fluorescent Protein (cYFP) fragment together with MPK3/4/6/8/11-nYFP. Positive interaction is indicated as fluorescence signal in the YFP channel; *Chl* chlorophyll autofluorescence, *bright* bright field, *merge* channel overlay. *Scale bars* 10 µm. **c** Western blot analyses of samples from **b** to monitor expression of the BiFC constructs. Samples were ran on duplicate gels for separate detection of MPKs (α-cMyc) and PRP/PH1/PH2 (α-HA). Membranes were stained with amido black to show equal loading (based on the staining of the Rubisco large subunit band). Experiments were performed three times with similar results

Querying the* Arabidopsis* interactome databases (http://bar.utoronto.ca/interactions/cgi-bin/arabidopsis_interactions_viewer.cgi) indicates that PH2 interacts with MPK3 and MPK6. To validate this prediction and the data from the initial Y2H screen, the full length ORFs of PRP, PH1 and PH2 were cloned for a detailed Y2H analysis against all the twenty *Arabidopsis* MAPKs. All three proteins interacted with MPK6, PRP and PH2 also interacted with MPK3 and MPK4, and PH2 additionally interacted with MPK11 (Fig. 2a; Supplemental Fig. 2). Notably, all these MAPKs were shown to be involved in defense signaling (Rasmussen et al. 2012). For PH1, additional interaction with MPK8 was detected, which is involved in reactive oxygen species (ROS) homeostasis in *Arabidopsis* (Takahashi et al. 2011). To verify these data *in planta*, bimolecular fluorescence complementation (BiFC) experiments were performed in *Arabidopsis* mesophyll protoplasts transiently co-expressing PRP, PH1 or PH2 fused to the C-terminal half of Yellow Fluorescent Protein (cYFP) and with MAPKs fused to the N-terminal half of YFP (nYFP). Again, as indicated by reconstituted YFP fluorescence within the cells, all proteins interacted with MPK6. For PRP and PH2, additional interaction with MPK3, MPK4 and MPK11 was observed, whereas neither PRP, PH1, nor PH2 interacted with MPK8 (Fig. 2b). To confirm expression of the proteins, western blots were performed with aliquots of the samples used for microscopy (Fig. 2c). Taken together, the interaction of PRP and its homologs with defense-related MAPKs could be confirmed with two different approaches. Differences in specificity observed in Y2H and BiFC experiments are most likely attributable to the different experimental systems.

### PRP, PH1 and PH2 are phosphorylated by MAPKs in vitro

**Fig. 3:**
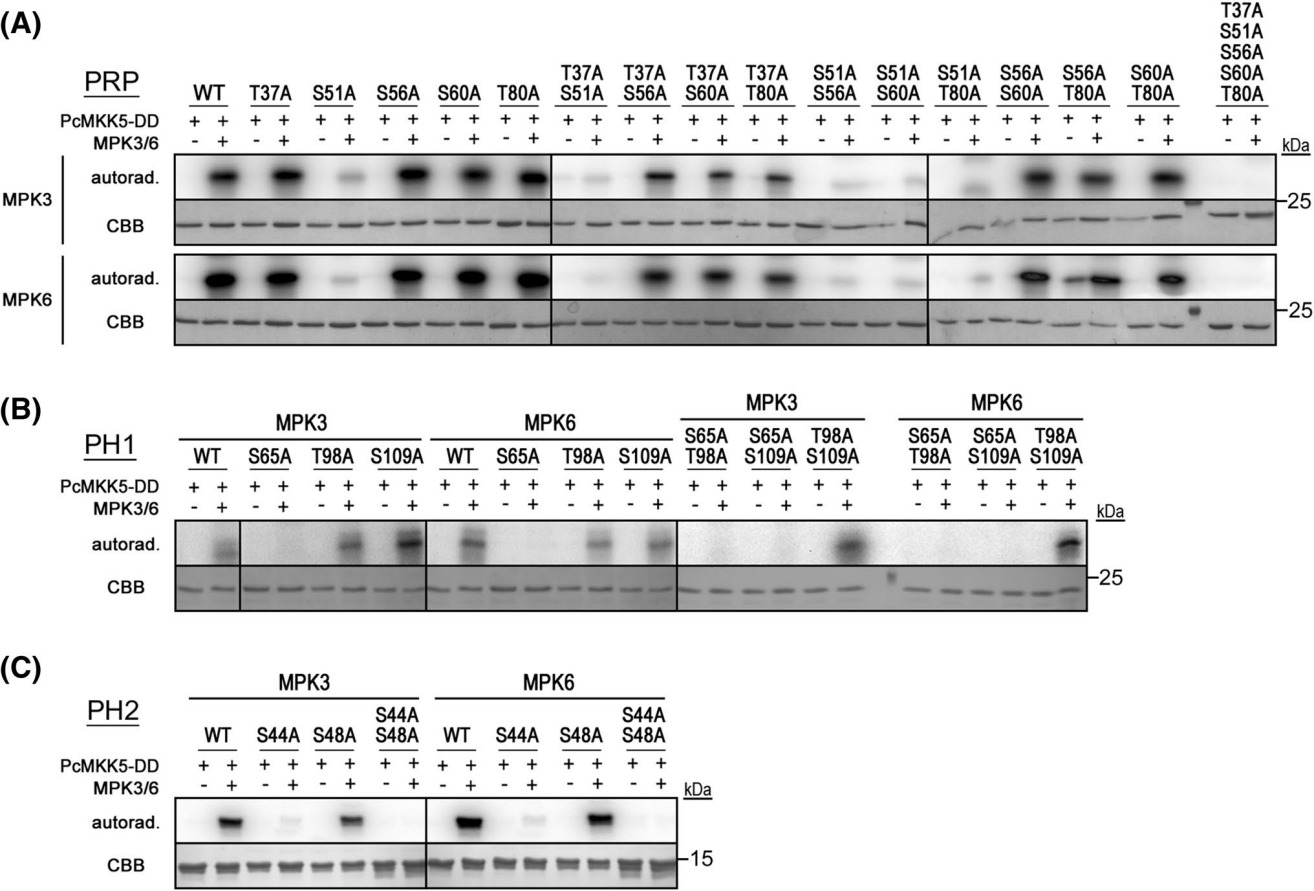
PRP, PH1 and PH2 are in vitro substrates of MPK3/6. **a**. Radioactive in vitro kinase assays with recombinant PRP wild type (WT) or putative MAPK phosphosite mutant proteins (individual, double and penta mutants) and MPK3/6. Activation of MPK3/6 was achieved in the presence of constitutively active parsley PcMKK5-DD (Lee et al. 2004). Proteins were incubated with radioactive ATP, separated by SDS-PAGE and analyzed by autoradiography (autorad.); *CBB* Coomassie Brilliant Blue stain (of the purified recombinant protein). Position of the protein size marker is indicated on the *right*. **b** and **c** Radioactive in vitro kinase assays with recombinant PH1/PH2 wild type (WT) or putative MAPK phosphosite mutant proteins (individual and double mutants) and MPK3/6. Samples were analyzed as in **a**. (This figure is previously part of Fig. 1d of the Palm-Forster et al. 2012 paper describing the mutagenesis method and is reprinted here with permission from Elsevier)

Since MAPKs target “[S/T]P” motifs (i.e. serine or threonine that precede a proline residue) and all three PRP-like proteins are rich in serine and proline, they are potential MAPK substrates. PRP, PH1 and PH2 possess five, three and two putative MAPK phosphosite respectively (Fig. 1a). We therefore addressed whether PRP and its homologs could be phosphorylated by MAPKs and aimed to identify the targeted phosphosites. Towards this end, we generated several versions of individual, double, and in the case of PRP, a penta phosphosite mutant(s) through a type IIs restriction enzyme-based mutagenesis method designed to rapidly mutate typical MAPK-type [S/T]P motifs to AP (Eschen-Lippold et al. 2014; Palm-Forster et al. 2012). All of the constructs were then expressed in *Escherichia coli* with an N-terminal His-tag for protein purification. All three proteins exhibited slower migration in SDS–PAGE compared to their calculated molecular weight. Furthermore, a second (presumably dimerized) form that is recalcitrant to SDS-denaturation existed for the recombinant PRP or PH1. As mass spectrometry analysis did not reveal post-translational modifications or peptides from other proteins (not shown), we attribute this to aberrant mobility of the proteins. Purified recombinant proteins were then used for radioactive in vitro kinase assays with PRP, PH1 or PH2 (as substrates) together with either GST-tagged MPK3 or MPK6 in the presence of PcMKK5-DD (a constitutively active version of a parsley MKK5) (Lee et al. 2004) to activate MPK3/6. Wildtype PRP, PH1 and PH2 were all phosphorylated by MPK3 and MPK6 (Fig. 3a–c). Whenever S51 (in PRP), S65 (in PH1) or S44 (in PH2) is mutated in the single or in higher order mutants, phosphorylation of the recombinant protein is strongly reduced (Fig. 3a–c), suggesting it to be the predominant site targeted by the MAPKs. Interestingly, this major phosphosite is conserved between all three proteins (*cf*. Fig. 1a).

### PRP, PH1 and PH2 carry a functional MAPK docking site

**Fig. 4:**
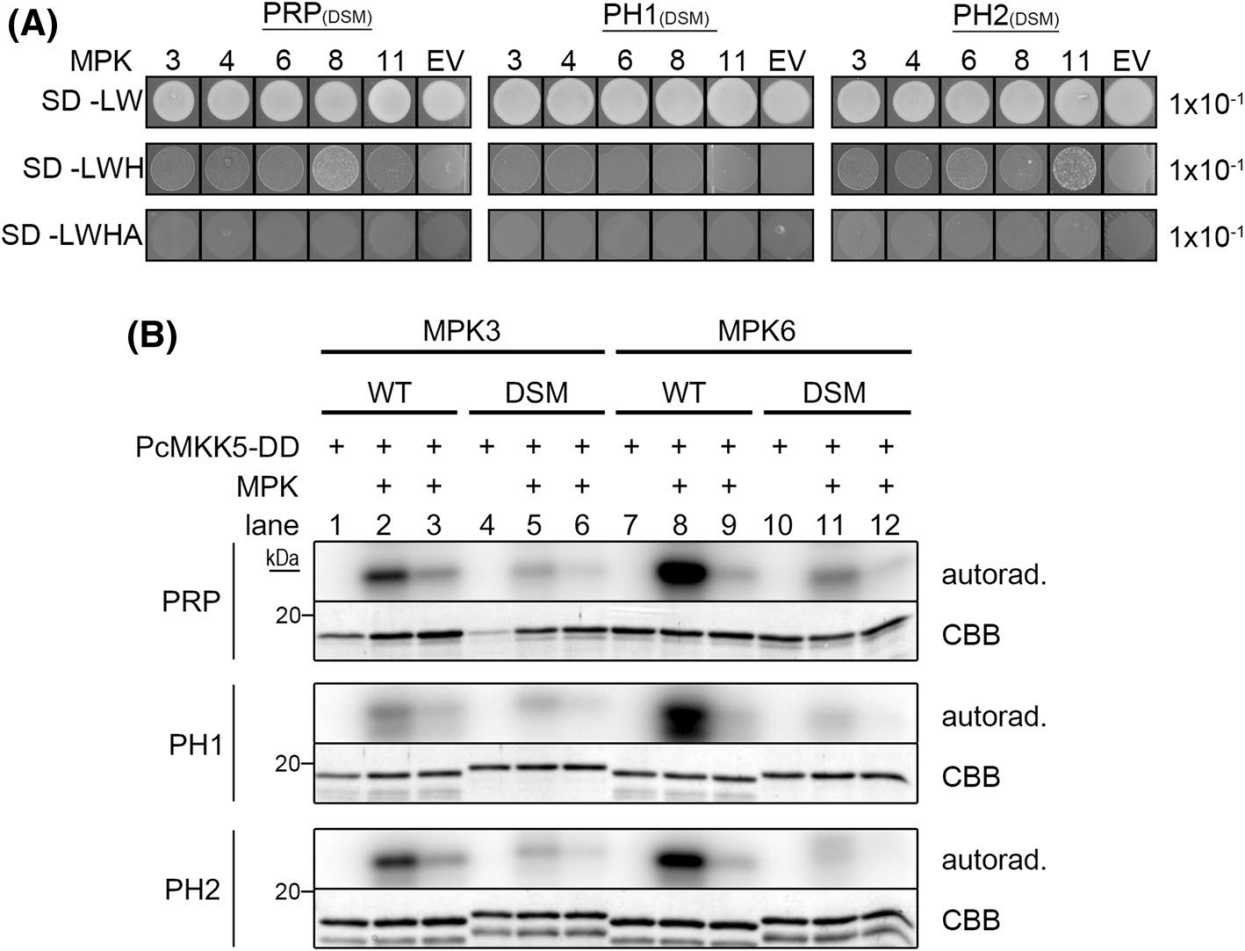
PRP, PH1 and PH2 have a functional MAPK docking site. **a** Yeast two-Hybrid (Y2H) interaction analyses of *Arabidopsis* MAPKs with PRP, PH1 and PH2 MAPK docking site mutant (DSM) versions (PRP: K26E, R27E, L31D, I33D; PH1: R42E, R43E, L47D, I49D; PH2: R21E, R22E, L27D, I29D). Positive interactions are indicated by yeast growth on selective synthetic drop-out media (SD-Leu/-Trp/-His or SD-Leu/-Trp/-His/-Ade; *cf*. Fig. 1a); *EV* empty vector control (pDEST32). **b** Radioactive in vitro kinase assays with recombinant PRP, PH1 and PH2 wild type (WT) or MAPK docking site mutant proteins (DSM) and either MPK3 or MPK6. Activation of MPK3/6 was achieved in the presence of constitutively active parsley PcMKK5-DD (Lee et al. 2004). Samples were analyzed as in Fig. 3a. For* lanes 3, 6, 9 and 12*, only 1/5 of MPK3/6 was used in comparison to* lanes 2, 5, 8 and 11*, respectively. *Autorad*. autoradiography, *CBB* Coomassie Brilliant Blue (staining of the recombinant substrate proteins)

**Fig. 5:**
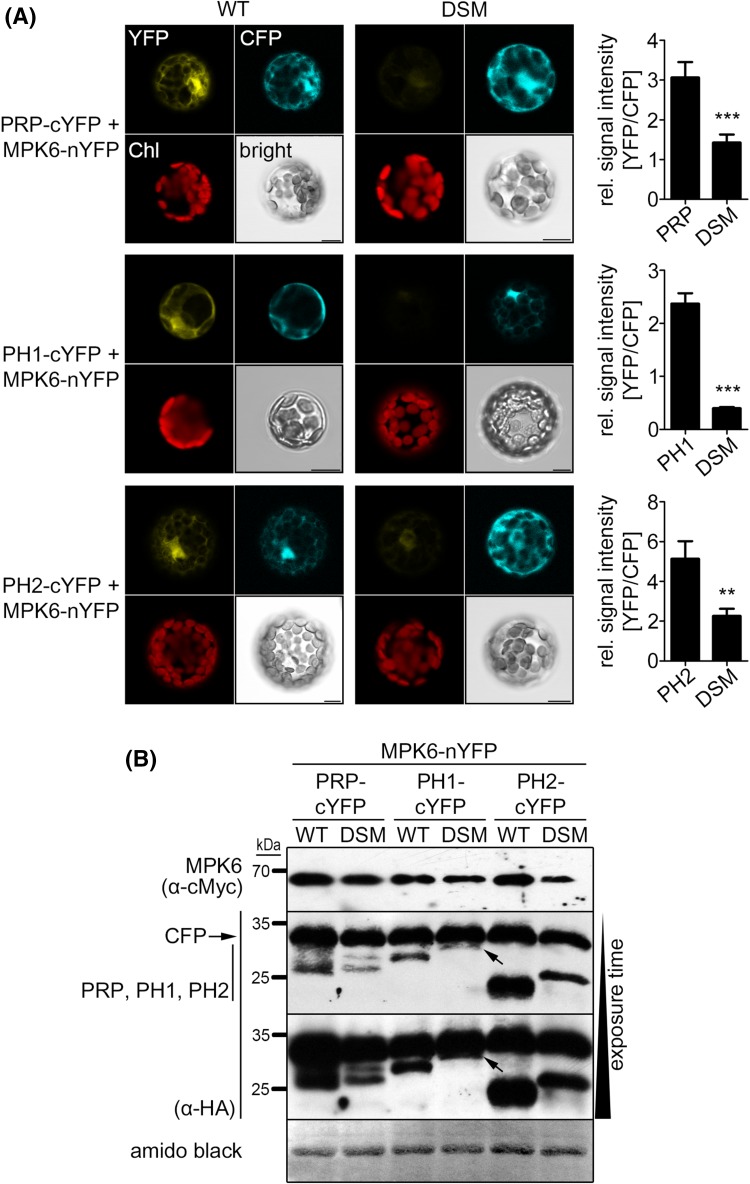
PRP, PH1 and PH2 MAPK docking site mutants are impaired in MPK-interaction in vivo. **a** Bimolecular fluorescence complementation (BiFC) assay in* Arabidopsis* protoplasts transiently co-expressing cYFP fusions of PRP, PH1 or PH2 wild type (WT) or MAPK docking site mutants (DSM) together with MPK6-nYFP plus CFP-HA. Positive interaction is indicated as fluorescence signal in the YFP channel; CFP = CFP channel (for normalization of fluorescence levels); *Chl* chlorophyll autofluorescence; *bright* bright field. *Scale bars* 10 µm. As a measure for interaction, fluorescence ratios (YFP/CFP) were calculated for each individual protoplast (*bar* charts next to the microscopy images; n ≥ 35). **b** Western blot analyses of samples from **a** to monitor expression of the constructs. The blot was subsequently probed with α-cMyc (MPK6) and α-HA (PRP/PH1/PH2/CFP) antibodies. (Note that for unknown reasons, the DSM PH1 and PH2 show aberrant gel mobility compared to the non-mutated proteins; this is also seen with the recombinant proteins, see Fig. 4b). The membrane was stained with amido black to show equal loading. Experiments were repeated twice with similar results

In silico sequence analyses of PRP and its homologs revealed the presence of putative MAPK docking sites with the consensus sequence (R/K)_1−2_-(X)_2−6_-Φ-X-Φ (where Φ represents any hydrophobic residue; Fig. 1a). Furthermore, prolines were found within and after the docking site, as was predicted for the refined consensus of plant MAPK substrate docking motif (Pitzschke 2015). The basic residues of the MAPK docking site are predicted to bind a negatively charged area of the respective kinase that is located C-terminally of its kinase domain, while the hydrophobic residues of the MAPK docking site bind within a conserved hydrophobic groove present in MAPKs (Ubersax and Ferrell 2007). To test the importance of the predicted MAPK docking sites within PRP, PH1 and PH2 for MAPK interaction and phosphorylation, mutant versions were generated. Basic residues (Lys/Arg) were exchanged against acidic Glu and hydrophobic residues (Leu/Ile) against acidic Asp (see Fig. 1a). In Y2H experiments, these mutations completely abolished the interaction with MAPKs (Fig. 4a; *cf*. Fig. 2a). In accordance to the reduced protein–protein interaction, phosphorylation of the mutant versions in in vitro kinase assays was also highly reduced compared to the wildtype proteins (Fig. 4b). To determine the effect of the MAPK docking site mutations on in vivo interaction with MAPKs, BiFC experiments were performed in mesophyll protoplasts. Wild type and mutant versions of PRP-, PH1- and PH2-cYFP were co-expressed with MPK6-nYFP, as well as HA-tagged CFP for normalization purposes. Strong reconstituted YFP signals indicated interaction between the wild type versions of PRP and its homologs with MPK6, whereas only weak signals could be detected in case of the MAPK docking site mutants (Fig. 5a). To compare the interaction strengths quantitatively, the ratios of measured signal intensities of YFP and CFP for each individual protoplast were calculated. The MAPK docking site mutants of PRP, PH1 and PH2, all showed significantly reduced values in comparison to their respective wild types (Fig. 5a, right panel). Western blot analysis showed that all the proteins were expressed at the expected sizes (Fig. 5b), so that the difference in reconstituted YFP signals is not due to the lack of protein accumulation.

### Flg22-induced degradation of PRP, PH1 and PH2

**Fig. 6:**
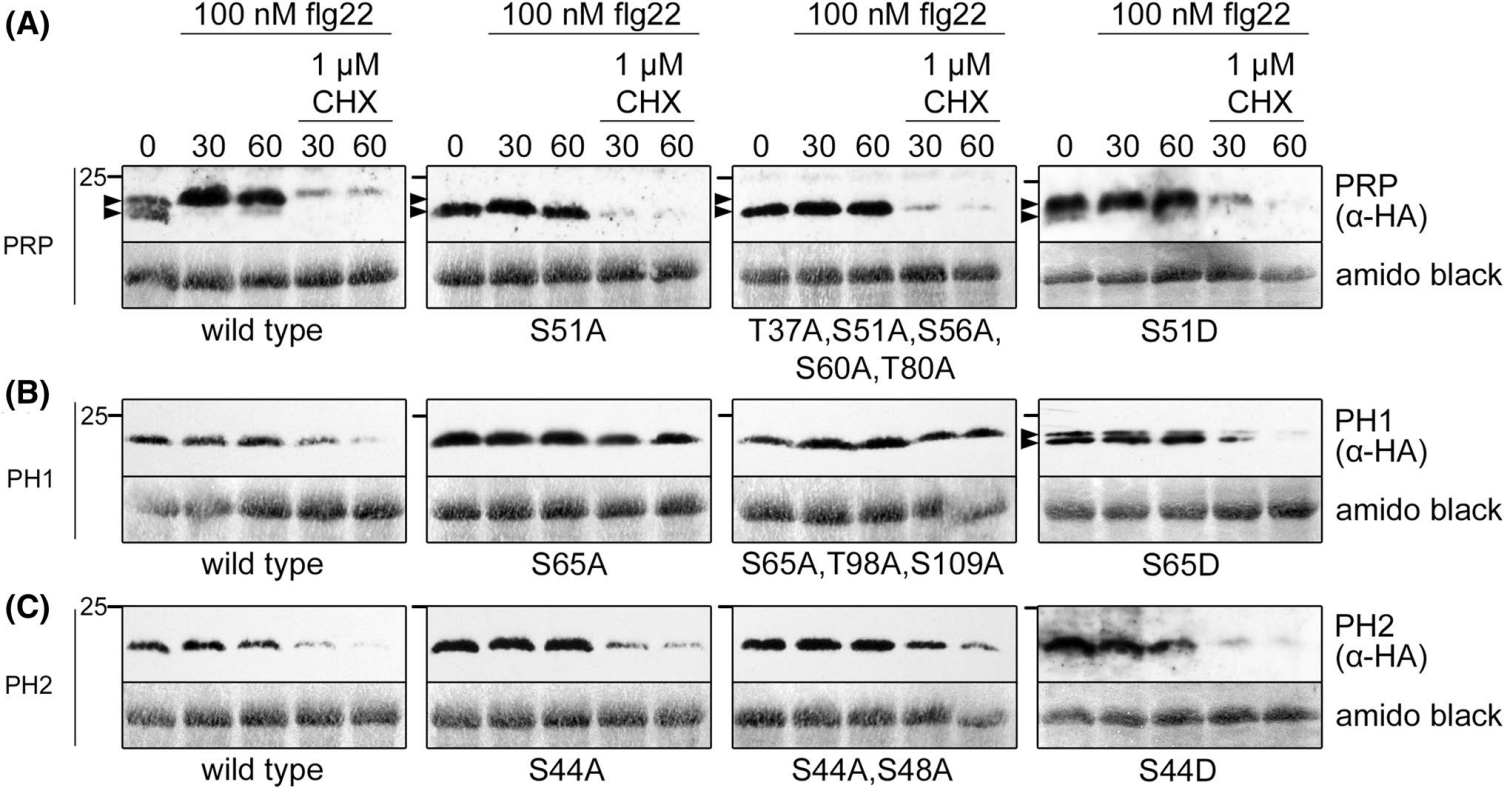
PRP, PH1 and PH2 show a phospho-shift and/or destabilization upon phosphorylation. **a–c** PRP, PH1 and PH2 wild type, phosphosite mutant (alanine substitutions) or phospho-mimic (aspartic acid substitutions) proteins were transiently expressed in protoplasts. Upon elicitation with 100 nM flg22 alone or in combination with 1 µM cycloheximide (CHX), samples were harvested at the indicated time points and subjected to Western blot analyses using an α-HA antibody. *Arrowheads* indicate a phospho-shift of wild type PRP and PRP-S51D, as well as a double band of PH1-S65D. Note that PRP phosphosite mutants lost the phospho-shift upon flg22 treatment; *mpt* minutes post treatment. Membranes were stained with amido black to show equal loading. Position of a 25-kDa marker protein is indicated on the *left* of each blot

Phosphorylation of MAPK substrate proteins often influences their stability or turn-over rates. For 1-aminocyclopropane-1-carboxylic acid synthase 6 (ACS6), the rate-limiting enzyme in ethylene biosynthesis, enhanced stability/protein accumulation was detected upon phosphorylation by MPK6 (Liu and Zhang 2004). By contrast, decreased stability was observed upon MAPK-mediated phosphorylation for other MAPK substrates, e.g. TZF9 or WRKY46 (Maldonado-Bonilla et al. 2014; Sheikh et al. 2016). In the case of PRP and its homologs, we compared wild type proteins with variants mutated in their primary phosphosite (see Fig. 1), mutated in all the potential phosphosites or phospho-mimic versions of the main phosphosites. To improve visualization of protein turn-over, which may otherwise be masked by the strong expression driven by the strong cauliflower mosaic virus (CaMV) 35S promoter, cycloheximide was added to block protein translation. Under these conditions, flg22-induced degradation could be seen on western blot level for all three wild type proteins (Fig. 6a–c). In addition, a flg22-induced mobility shift could also be detected for the native PRP, which apparently is phosphorylation-dependent since the phosphosite mutants (S51A and penta mutant) were not shifted upon flg22 treatment, whereas the phospho-mimic version (S51D) showed a slightly enhanced shift. However, altered protein stability was not detected for the PRP variants, indicating that phosphorylation of PRP is probably not involved in degradation or enhanced stability (Fig. 6a). On the contrary, the PH1 phosphosite mutants (S65A and triple mutant) were more stable compared to the wild type protein, pointing to phosphorylation-dependent destabilization. Interestingly, the phospho-mimic version (S65D) was detected as a double band (Fig. 6b). In case of PH2, the double phosphosite mutant showed some enhanced stability compared to the wild type, indicating phosphorylation-dependent degradation similar to PH1 (Fig. 6c). According to these data, phospho-mimic versions of PH1 (S65D) and PH2 (S44D) should be less stable than the respective wild types but this was not the case. It is possible that the aspartate mutations were inefficient as phospho-mimics in comparison to the authentic phospho-modification of these proteins.

### Expression and subcellular localization of PRP, PH1 and PH2

**Fig. 7:**
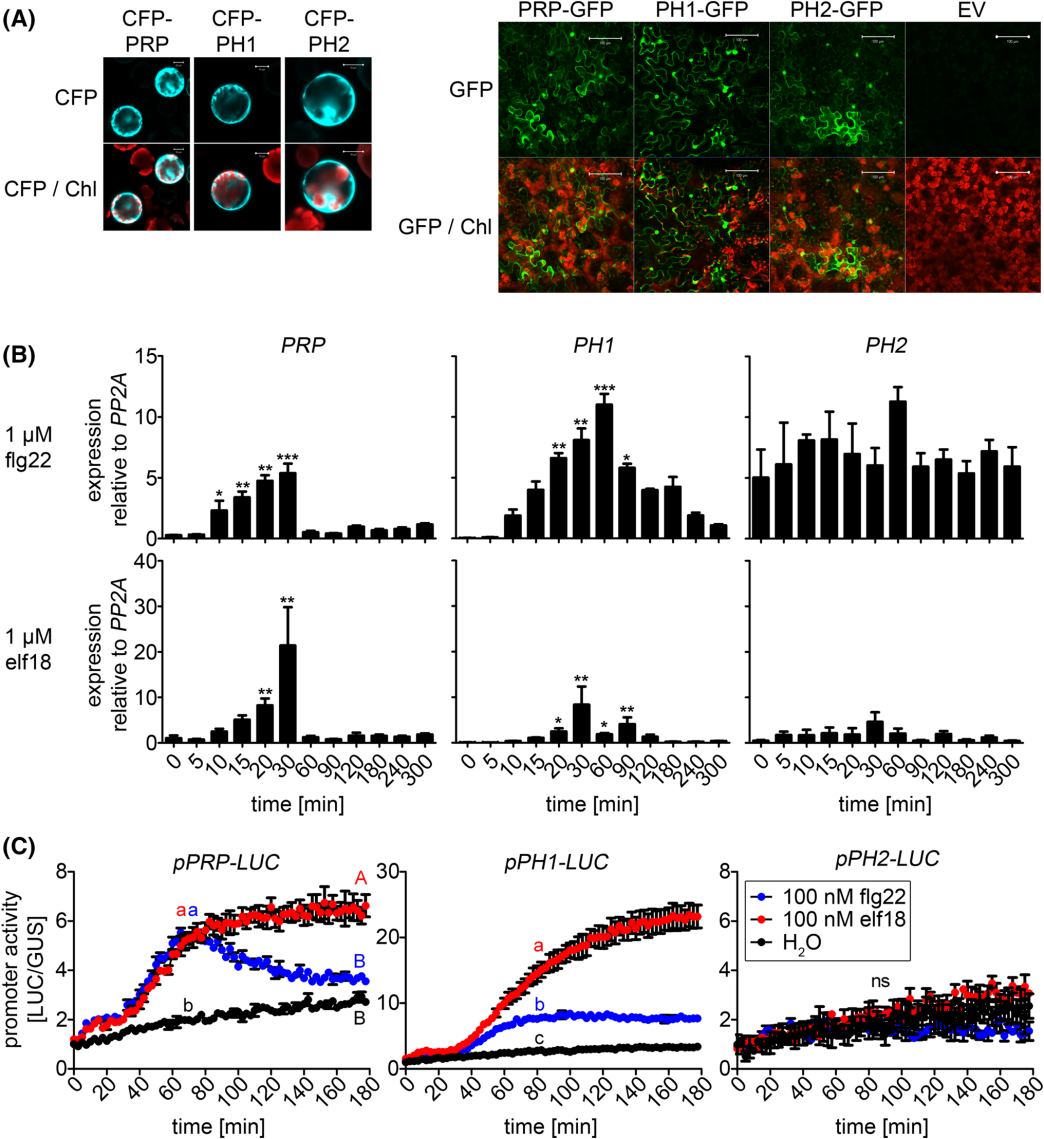
Expression and subcellular localization of PRP, PH1 and PH2. **a** Transient expression of PRP, PH1 and PH2 constructs tagged with N-terminal CFP or C-terminal GFP in mesophyll protoplasts (*left* panel) or *Nicotiana benthamiana* (*right* panel) for subcellular localization. Photos of CFP/GFP and chlorophyll autofluorescence (Chl) channels were taken. An empty vector construct (EV) served as a negative control. *Scale bars* 10 µm (*left* panel)/100 µM (*right* panel). **b** Expression analyses of PRP and its homologs in seedlings treated with the PAMPs, flg22 and elf18. Seedlings were grown in liquid culture for 2 weeks and treated with 1 µM flg22/elf18. Samples were harvested at the indicated time points. Total RNA was isolated, reverse transcribed and used for quantitative realtime PCR to measure *PRP, PH1* and *PH2* transcript levels (relative to *PP2A* as a reference gene). Asterisks indicate statistically significant differences compared to [t = 0 minutes] (n = 3; Kruskal–Wallis One-way ANOVA with Dunns posttest; *p < 0.05; **p < 0.01; ***p < 0.001). The experiment was repeated twice with similar results. **c** Luciferase assays in mesophyll protoplasts using *PRP*/*PH1*/*PH2* promoter-luciferase fusions as reporter constructs. Luciferase-mediated light emission was recorded for 3 h post PAMP treatment (100 nM flg22/elf18) as a measure for promoter activity. For normalization, a *pUBQ10-GUS* construct was co-transformed. LUC/GUS ratios were calculated and fold changes relative to the respective water control [t = 0 minutes] were plotted. Letters indicate statistically significant differences (n = 3; Two-way RM ANOVA with Bonferroni posttests; p < 0.01; *ns* not significant). The experiment was performed four times with similar results

To get some idea of their putative functions, the expression pattern and subcellular localization of PRP and its homologs were analyzed. According to publicly available gene expression data, PRP is highly expressed in stamen and particularly in pollen. Quantitative RT-PCR confirmed the high expression in pollen and also the moderate expression in flowers and siliques (Supplemental Fig. 3). Comparatively, PH1 and PH2 are expressed at much lower levels in all tissues, with some expression detected in mature siliques. For the subcellular localization of PRP and its homologs, we generated constructs with N-terminal CFP or C-terminal GFP tags to largely rule out any adverse effects based on the position of the tag. Transient expression experiments in both* Arabidopsis* mesophyll protoplasts and *Nicotiana benthamiana* (*Agrobacterium tumefaciens*-mediated delivery) revealed cytoplasmic and nuclear localization of PRP, PH1 and PH2 (Fig. 7a), similar to the data obtained in the BiFC experiments (Fig. 2b).

Since all three proteins interacted with defense/stress-related MAPKs in Y2H and BiFC experiments (Fig. 2a, b), we analyzed their expression upon application of various abiotic stresses and PAMP treatments in whole seedlings by quantitative RT-PCR. Cold, heat, salt or drought treatments did not induce significant changes in transcript levels of *PRP, PH1* and *PH2* (Supplemental Fig. 4). On the other hand, elicitation with the PAMPs, flg22 and elf18, significantly induced transient transcript accumulation of *PRP* and *PH1*, whereas *PH2* transcript levels did not change (Fig. 7b). Similarly, in a luciferase reporter assay using *PRP, PH1* and *PH2* promoters to drive luciferase expression, only the *PRP* and *PH1* promoters, but not the *PH2* promoter, were significantly activated upon both flg22 and elf18 treatment resulting in elevated luciferase expression and activity (Fig. 7c). These findings imply that *PRP* and *PH1* are transcriptionally activated upon flg22 or elf18 elicitation. In agreement with this, in silico expression datamining shows inducible expression of *PRP* and *PH1* by additional PAMPs such as chitin, NPP1 or hrpZ harpin (Hruz et al. 2008).

### Over-expression of PRP, PH1 and PH2 influences plant development

**Fig. 8:**
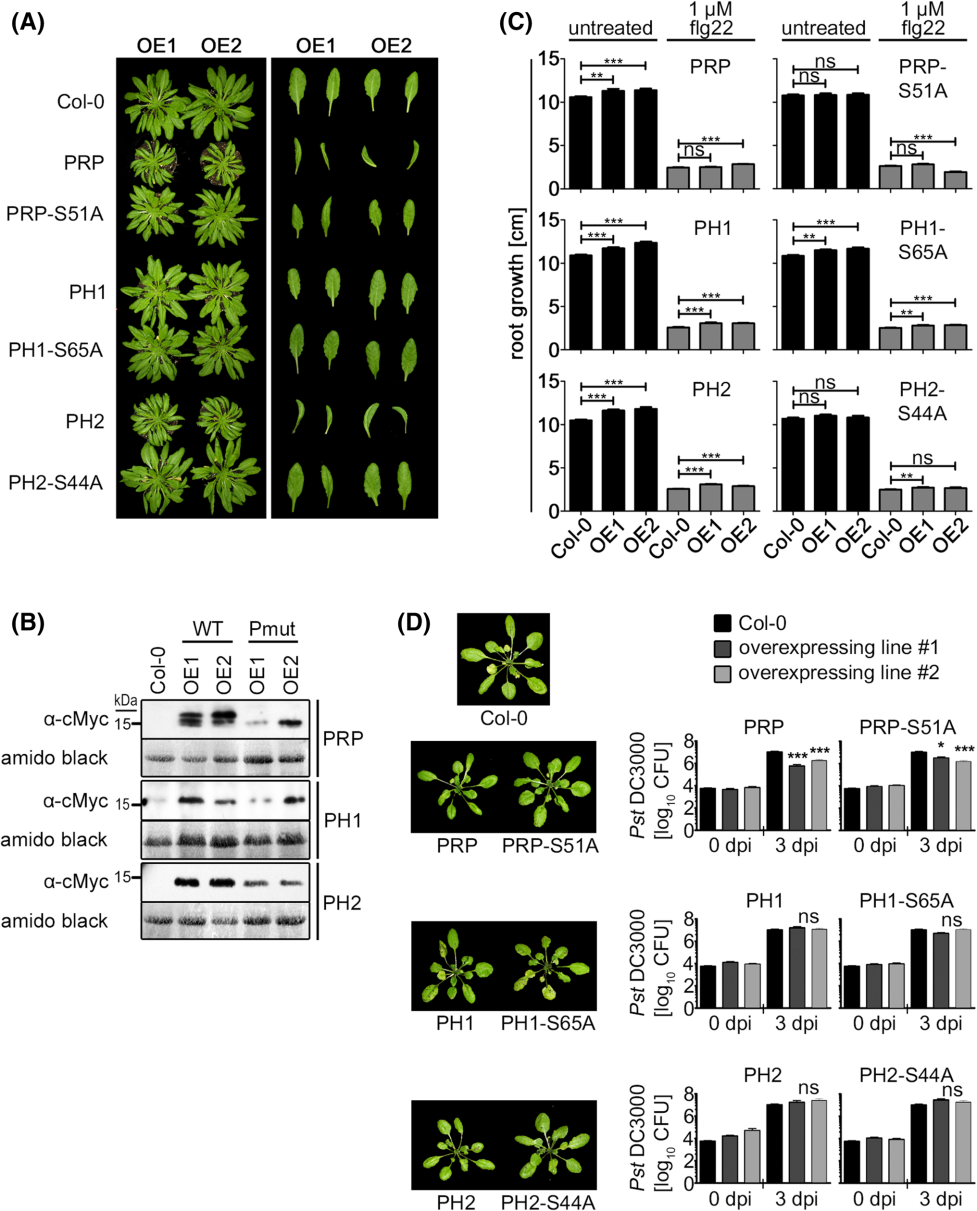
Characterization of transgenic plants over-expressing wild type or phosphosite mutant versions of PRP, PH1 and PH2. **a** Growth phenotype of plants grown for 8 weeks on soil under short day conditions (8 h light/16 h darkness, 22 °C). The *right panel* shows individual adult leaves. Two independent over-expressing *lines* per genotype are shown (OE1/OE2). Note that only the main individual phosphosites were mutated in the phosphosite mutants (PRP-S51A, PH1-S65A, PH2-S44A; *cf*. Fig. 2). **b** Western blot (α-cMyc) showing expression of the constructs in the transgenic lines in **a**. Membranes were stained with amido black to show equal loading (*WT* wild type, *Pmut* phosphosite mutants). **c** Root growth inhibition assays with seedlings grown on plates ±1 µM flg22. The experiment was performed twice with similar results and the combined data are shown. *Asterisks* indicate statistically significant differences (Mann–Whitney test; **p < 0.01; ***p < 0.001). **d** Infection assay with *Pseudomonas syringae* pv. *tomato* (*Pst*) DC3000. Plants were grown for 3 weeks on soil under short day conditions (8 h light/16 h darkness) and then spray-inoculated with a bacterial solution (5 × 10^8^ cells/ml). Bacterial growth was determined (0 and 3 dpi) by counting colony-forming units (CFU) after plating serial dilutions. The experiment was performed 5 times with similar results (the diagram shows combined data sets; n = 15). *Asterisks* indicate statistically significant differences at day 3 between over-expressing lines and the wild type (Col-0; Mann–Whitney test; *p < 0.05; ***p < 0.001; *ns* not significant). On the *left* side, photos of infected plants at day 3 are shown

Stable transgenic lines over-expressing cMyc-tagged forms of either wild type PRP, PH1 and PH2 or the main phosphosite mutant versions PRP-S51A, PH1-S65A and PH2-S65A under the constitutive CaMV-35S promoter were generated by *Agrobacterium*-mediated transformation. Two transgenic lines were selected for each construct for further characterization. Within the first 4 weeks of growth on soil under short day conditions, no obvious differences to the wild type Col-0 accession were observed. However, in older plants over-expressing wild type PRP, the rosette diameters were smaller and leaves were curled inward. Plants with the phosphosite mutant PRP-S51A did not show this phenotype (Fig. 8a). PH1 wild type and phosphosite mutant over-expressing lines (PH1-S65A) did not show any clear difference compared to Col-0 (Fig. 8a). PH2 wild type over-expressing plants looked similar to PRP over-expressors with smaller rosettes and inward curled leaves, whereas the phosphosite mutant lines (PH2-S44A) were indistinguishable from Col-0 (Fig. 8a). To demonstrate expression of the proteins, leaf material was harvested and subjected to α-cMyc Western blot analyses (Fig. 8b).

Since over-expression of PRP and PH2 has an influence on leaf development, we next assessed whether root growth was affected in seedlings grown on agar plates. Under control conditions, PRP, PH1 and PH2 wild type over-expressors all developed slightly longer roots than the Col-0 control plants (Fig. 8c). In case of the phosphosite mutants, only PH1-S65A-expressing lines showed slightly enhanced root growth, for PRP-S51A and PH1-S44A no difference was detected. Thus, on root growth level, the effect is opposite to the leaf/rosette development phenotype with respect to the genotype (Fig. 8a, c). It is well documented that seedlings are retarded in development, including root growth, when continuously exposed to PAMPs (Gómez-Gómez et al. 1999). To analyze the influence of PRP, PH1 and PH2 over-expression on PAMP-mediated root growth inhibition, we included flg22-supplemented agar-plates in the analyses. Generally, a strongly reduced root growth was measured compared to control conditions, but differences between genotypes were less pronounced. Based on the statistics, only PH1 and PH2 wild type, as well as PH1-S65A-expressing lines developed marginally longer roots compared to Col-0 (Fig. 8c). For the other lines, inconsistent results were obtained between the two tested transgenic lines, so that no clear conclusions can be drawn.

### PRP and PRP-S51A over-expression enhances resistance against *P. syringae* pv. *tomato* DC3000

To assess the impact of over-expression of PRP and its homologs on resistance against pathogens, inoculation experiments were performed with the transgenic lines and the bacterial pathogen *P. syringae* pv. *tomato* (*Pst*) DC3000, which is able to infect and multiply in* Arabidopsis* (Katagiri et al. 2002). To minimize any adverse influences of the growth phenotype of PRP, PRP-S51A and PH2 over-expressing lines, young plants (3 weeks old) with no obvious difference in morphology were used. Plants were spray-inoculated and bacterial growth was assessed. Only PRP and PRP-S51A over-expressing lines were significantly more resistant than the Col-0 wild type plants; while no statistically significant differences to Col-0 were observed for plants over-expressing either PH1 or PH2 (Fig. 8d). This suggests that PRP, PH1 and PH2 have distinct roles in regulating resistance.

### ROS homeostasis is affected in the PRP-OE plants

We wondered if resistance to *Pst* conferred by PRP-overexpression might be linked to feedback signaling on MAPK functions. However, in three independent experiments, we did not observe any consistent changes in the flg22-induced MAPK activation profile in the PRP-overexpressing lines (Fig. S5). More recent survey of *PRP* expression pattern hinted of a connection to ROS signaling (see discussion below). We thus also looked at activation of MAPKs by H_2_O_2_ treatment but saw no differences between Col-0 wild-type plants compared to the PRP-overexpressing lines (Fig. S5). Taken together with the observation that enhanced resistance to *Pst* is obtained by overexpressing either the native PRP or the non-phosphorylatable S51A variant, the PRP effect on resistance is likely downstream (or independent) of MAPK activities.

**Fig. 9:**
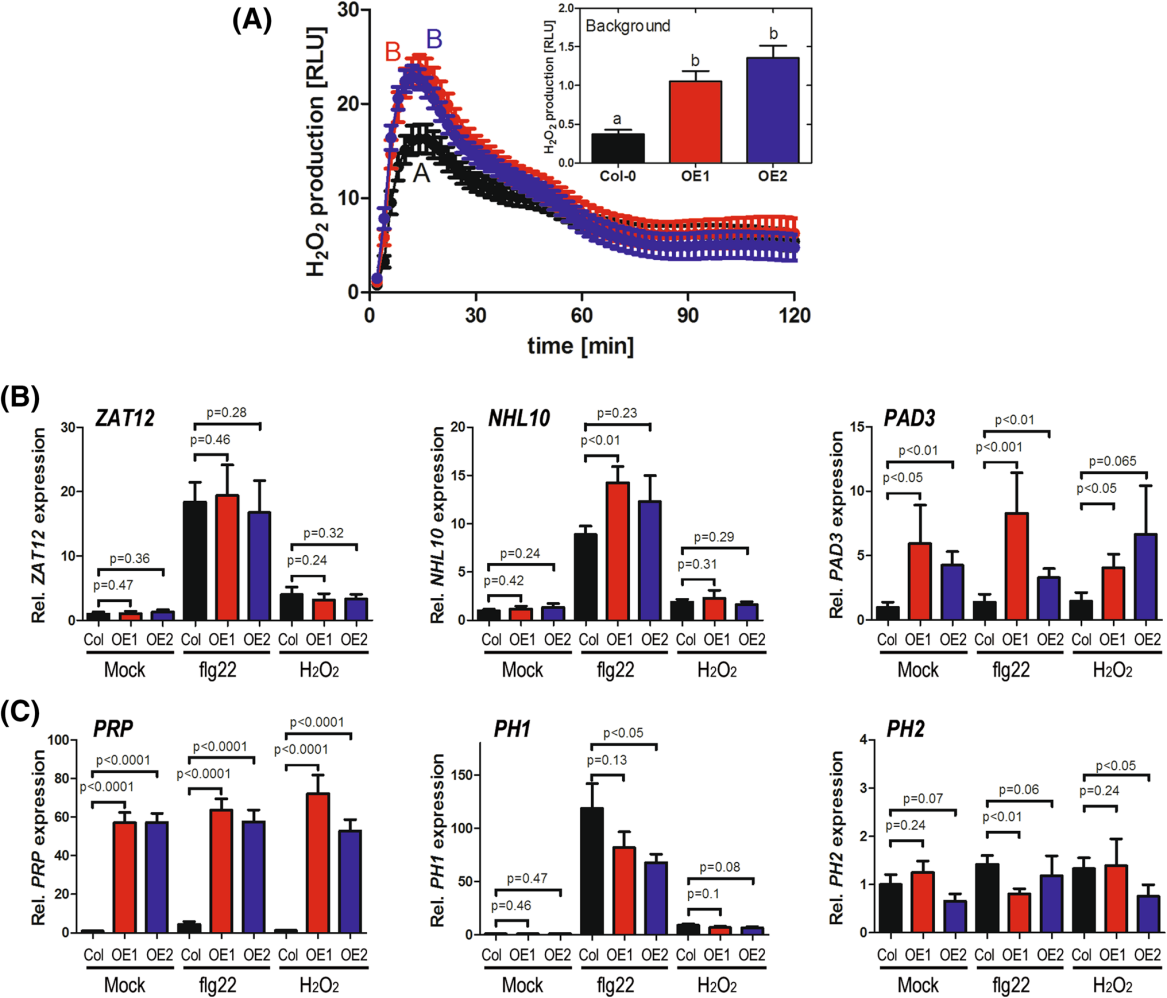
ROS accumulation and *PAD3* expression are enhanced in the PRP-overexpressors. **a** Flg22-induced H_2_O_2_ accumulation was quantified using a luminol-based assay. Four independent experiments were performed with different batches of plants, each with 24 leaf discs (*Black* = Col-0; *red*/*blue* = PRP overexpressor *line 1* and *2*, respectively). For the depicted graph, data were pooled from all four experiments (n = 4 × 24 leaf discs) and reported as the mean relative light units (RLU). Error bars are the standard errors. *Inset* shows the basal ROS levels prior to flg22 treatment. *Different alphabets* mark statistically distinct (ANOVA) groups. **b** qRT-PCR showing expression of the indicated defense-related genes. Flg22 (100 nM), H_2_O_2_ (2 mM) or water (mock) treatments were performed for 30 min and the RNA extracted for RT-PCR analysis. Data is pooled (n = 9) from three independently performed experiments (each with 3 replicates) and shown as relative expression levels (normalized to the average of the Col-0/mock-treated sample). For statistical significance test, the data was log_2_-transformed and the *p*-values of the pair-wise *t* test comparison (to the corresponding Col-0 genotype) are shown above each *bar*. **c** Expression of *PRP, PH1* and *PH2* was analyzed as in “**b**” above

Due to the possible link to ROS signaling, we next measured ROS levels and observed higher H_2_O_2_ levels in the flg22-stimulated leaf discs taken from the PRP-overexpressing lines. To validate this, the experiment was repeated and the same result was consistent in four times independently (Fig. 9a). Closer inspection also revealed that the basal ROS levels prior to flg22 treatment was already elevated (see insert in Fig. 9a). However, expression of a ROS-responsive marker gene, *ZAT12* (Davletova et al. 2005), was not raised in the PRP-overexpressors, neither in the mock nor the flg22/H_2_O_2_-treated samples (Fig. 9b). Thus, downstream ROS signaling does not appear to be strongly affected in the PRP-overexpressing lines.

To get an idea of how PRP-overexpression might confer resistance, we analyzed expression of a number of defense-related genes by quantitative RT-PCR. We found a tendency of higher flg22-induced expression of *NHL10* in the PRP-overexpressors, but statistical significance was obtained for only one of the two overexpressing lines (Fig. 9b). We validated our expression analysis in three independent repetitions and also confirmed that the lines used were indeed PRP-overexpressors (i.e. the transgene was not silenced) and did not have strong effects on expression of the related *PH1* or *PH2* genes (Fig. 9c). Among the tested genes, expression of *PAD3*, encoding a key enzyme in camalexin biosynthesis (Zhou et al. 1999), is consistently higher in the overexpressors compared to Col-0 controls (Fig. 9b, right panel).

## Discussion

### PRP, PH1 and PH2 are substrates of stress-activated MAPKs

We report here a previously undescribed family of MAPK-interacting proteins that are rich in proline and serine residues, small in size (11.3–14.1 kDa), and apparently specific to dicotyledonous plants. The most consistent interaction is observed with MPK6 for all three proteins in both Y2H and BiFC assays. Additional interactions with MPK3, MPK4 and MPK11 (as well as MPK8 for PH1) were also detected with either Y2H or BiFC. Since all 20* Arabidopsis* MAPK proteins were tested in the Y2H experiment but only five MAPKs were detected to interact in various combinations with PRP and its homologs (Fig. 2a; Supplemental Fig. 2), there is some specificity in the interaction. Interestingly, the interacting MAPKs (MPK3, MPK4, MPK6 and MPK11) are involved in pathogen defense signaling (Bethke et al. 2012; Rasmussen et al. 2012). Additionally, MPK3, MPK4 and MPK6 are also ascribed roles in abiotic stress signaling e.g. salt, cold, wounding and osmotic stress (Droillard et al. 2002; Teige et al. 2004), while MPK8 is associated with oxidative stress (Takahashi et al. 2011). Thus, this suggests that PRP and its homologs may play some role(s) in plant stress responses.

Corroborating the interaction analyses, kinase assays proved that recombinant PRP, PH1 and PH2 can be in vitro phosphorylated by MPK3 and MPK6 (Fig. 3), which is dependent on a conserved MAPK docking site. The major phosphosite targeted by MPK3 and MPK6 was also identified in all three proteins, where they differentially regulated in vivo protein stability in PRP, PH1 and PH2 (Fig. 6). With the exception of the L at the −2 position relative to the phosphorylated “SP” core, this major phosphosite did not fit the [LP]-[PX]-S-P- [RK] predicted consensus for MPK3/MPK6 substrates (Sorensson et al. 2012). This suggests that screens based on short 18-amino acid-peptides may not necessarily reflect *bona fide* MAPK target sites in intact proteins.

Since all the interaction analysis reported here are based on ectopic expression of the *PRP*s, a crucial question is whether there is overlapping expression with the relevant *MPK*s to allow in vivo phosphorylation in its endogenous, non-transgenic background. In particular, the eFP-browser depiction and qRT-PCR data (Fig. S3a) seem to suggest tissue-specific expression for some of the *PRP*-like genes (e.g. only in pollen for *PRP*). Based on the thousands of microarray experiments within the Genevestigator database, the high mRNA levels of *PRP* in pollen overlap with *MPK6* transcript levels (Fig S3d), and more importantly, the low but ubiquitous basal expression of *PRP, PH1* and *PH2* in leaves (the tissue used for most of the experiments in this work) is, in fact, comparable to or only slightly lower than *MPK3* and *MPK6* (Fig S3d). The nucleo-cytoplasmic sub-cellular localization of the PRPs (Fig. 7a) also overlaps with what is generally known for MAPKs. Therefore, while final confirmatory experiments with expression driven by native promoters will be invaluable, MPK3 or MPK6 probably can interact with and phosphorylate the three PRP-like proteins in their native expression context. Taken together with the findings that *PRP* and *PH1* expression is transcriptionally inducible by PAMPs (Fig. 7b, c), PRP and its homologs are novel MAPK substrate proteins that are potentially involved in defense- or stress-related signaling.

### PRP, PH1 and PH2 may be involved in plant development and/or stress response

A common strategy to uncover function of previously uncharacterized proteins is to study cellular localization. Unfortunately, the general cytoplasmic and nuclear localization did not provide further hints of its possible function. Bearing in mind the possible caveats of any influence from the tags used for visualization, there was also no specific localization to cell wall or extracellular matrix, which might be expected for some proline-rich proteins such as extensins (Kavi Kishor et al. 2015). The lack of T-DNA insertion mutants or homology to proteins with known function also hampered functional analysis. We therefore relied on the over-expression studies, where altered leaf development (for PRP and PH2) and root length (for all three proteins) were observed. As PRP is highly expressed in pollen and PH1/2 during embryo/seed development, the growth aberration upon over-expression suggests that they can interfere with and therefore may play some role in plant development.

Alternatively, the trade-offs between defense regulation and plant growth may account for the observed change in development upon over-expression. Plants must balance resources invested in growth and defense against pathogens, since both aspects are vital for survival and reproduction. Complex regulatory networks involving hormone signaling fine-tune this balance to adjust to changing environmental conditions and pathogen threats (Huot et al. 2014). When plants are grown under defense response-inducing conditions (e.g. growing in the presence of PAMPs), resources are channeled into pathways supporting defense. Here, due to constant defense signaling, root growth and development is negatively influenced (Gómez-Gómez et al. 1999; Zipfel et al. 2006). Interestingly, in PRP over-expressing plants, a similar inverse correlation between growth and defense could be observed. The plants are smaller in size but are more resistant to *Pst* DC3000 (Fig. 8a, d). PH2 over-expressing lines are similarly small, whereas the phosphosite mutant over-expression lines of both PRP and PH2 appear more like the Col-0 control plants (Fig. 8a). Notably, the conserved phosphosite in the PRP protein family (see Fig. 1a) is essential for the leaf development phenotype, thus implicating phospho-mediated regulation of PRP and PH2 function. As this is the major MAPK targeted site, MAPKs presumably are involved in this regulation in vivo.

The PH1 over-expressors did not show leaf developmental phenotype or enhanced disease resistance, which may be due to its extended N-terminus (Fig. 1a). Taken together with the different spatio-temporal gene expression patterns, these three members of this novel family of MAPK substrates presumably have distinct functions. The strong levels of *PRP* mRNAs in pollen suggest PRP is indicative of specific functions in pollen. However, no apparent differences in seed set was observed in the *PRP*-overexpressing lines but this may only become evident when single or higher order mutants of the *PRP* gene family are created (e.g. by CRISPR-Cas genome editing) in the future. With the current limited knowledge on this uncharacterized protein family, it is difficult to predict what their functions might be. Nevertheless, the PAMP-inducible expression of *PRP* and *PH1* suggests they may be involved in defense response upon pathogen attack. For PRP, this is supported by the enhanced resistance to *Pst* DC3000 conferred through *PRP* overexpression. Interestingly, *PRP* expression is elevated in transgenic plants with altered expression of an alternate oxidase gene (Umbach et al. 2005), as well as in a mutant of the *EXECUTOR1*/*2* genes (*ex1*/*ex2*) that encode two chloroplast-localized proteins involved in the retrograde control of nuclear gene expression by plastid signals during stress-induced release of singlet ^1^O_2_ (Lee et al. 2007). Furthermore, numerous microarray data show strong induction of *PRP* expression during hypoxia (Branco-Price et al. 2005). Therefore, *PRP* may be involved in ROS signaling. While considered to act as independent signaling pathways, MAPK and ROS are tightly connected (Kroj et al. 2003; Xu et al. 2014). Accumulation of ROS is rapidly induced after PAMP treatment (Kroj et al. 2003; Ranf et al. 2011) and ROS can also activate MAPKs (Pitzschke and Hirt 2006). Transient oxygen deprivation also leads to MPK3, MPK4 and MPK6 activation (Chang et al. 2012). Thus, PRP may be one of the MAPK substrates connecting MAPK signaling to downstream ROS responses. In this respect, pollen germination is affected in* Arabidopsis* mutants impaired in glutathione synthesis (e.g. *phytoalexin-deficient*, *pad2-1*), indicating that glutathione-mediated ROS detoxification may be essential for pollen development (Zechmann et al. 2011), where the high *PRP* expression in pollen may play a role. In agreement to these possible links between PRP and ROS signaling, H_2_O_2_ accumulation is elevated in the PRP-overexpressors. The enhanced *PAD3* expression in these plants is likely to be a direct corresponding effect since altered ROS levels (generated from perturbing chloroplast function or through methyl viologen application) are known to induce *PAD3* expression (Laloi et al. 2007; Nomura et al. 2012; Scarpeci et al. 2008). Whether the observed resistance conferred by overexpressing *PRP* is connected to altered ROS levels and *PAD3* expression remains to be determined.

In summary, tight regulation of signaling events is essential for optimal growth. MAPK cascades are important modules within complex signaling networks and represent central signal integration hubs linking different pathways. Their functions are executed ultimately by the pathway-specific substrates. We identified a new family of* Arabidopsis* MAPK substrate proteins that are potentially involved in both developmental and defense-related aspects. A prospective role in ROS homeostasis is likely in the case of the PRP protein. Future work will aim to elucidate the biochemical and molecular functions of these novel proteins.

## Materials and methods

### Plant growth conditions and treatments

All experimental work was performed with *Arabidopsis thaliana* (ecotype Col-0). Stable over-expression lines of wild type and phosphosite mutant versions of PRP, PH1 and PH2 were generated by the floral dip method (Logemann et al. 2006). Plants grown on soil under short-day conditions (8 h light/16 h darkness; 22 °C) in a phytochamber were used for protoplast assays and infection experiments. Seedling assays were performed under sterile conditions (long day regime: 16 h light/8 h darkness; 22 °C) with surface-sterilized and stratified seeds (4 °C for 2 days). For quantitative real time PCR experiments, seeds were germinated and grown in liquid culture (0.5× MS medium supplemented with 0.25% sucrose and 1 mM MES, pH 5.7) in microtiter plates. Seedlings were treated with PAMPs (1 µM flg22/elf18) for the indicated time points. For root growth assays, seeds were germinated and grown vertically on ATS-agar plates (Estelle and Somerville 1987) containing ±1 µM flg22. Localization studies in *Nicotiana benthamiana* were performed with plants grown in a greenhouse at 22 °C with 80% humidity.

### Phylogenetic analysis

The evolutionary history was inferred using the neighbor-joining method (Saitou and Nei 1987). The optimal tree with the sum of branch length = 1.40565089 is shown. The percentage of replicate trees in which the associated taxa clustered together in the bootstrap test (1000 replicates) are shown next to the branches (Felsenstein 1985). The tree is drawn to scale, with branch lengths in the same units as those of the evolutionary distances used to infer the phylogenetic tree. The evolutionary distances were computed using the Poisson correction method (Zuckerkandl and Pauling 1965) and are in the units of the number of amino acid substitutions per site. All positions containing gaps and missing data were eliminated from the dataset (Complete deletion option). There were a total of 81 positions in the final dataset. Phylogenetic analyses were conducted in MEGA4 (Tamura et al. 2007).

### Sequence alignment

PRP, PH1 and PH2 protein sequences were aligned using Clustal Omega [CLUSTAL O[1.2.1]; (Sievers et al. 2011)]. The alignment was exported in MSF format and run on the BoxShade server using the RTF_new output format (http://www.ch.embnet.org/software/BOX_form.html).

### Molecular cloning

PRP (At3g23170), PH1 (At4g14450) and PH2 (At1g04330) coding sequences were amplified from *A. thaliana* Col-0 cDNA and cloned into the pENTR^TM^/D-TOPO^®^ vector (Invitrogen). Correct clones were used to generate phosphosite, phospho-mimic and MAPK docking site mutants by site-directed mutagenesis involving type IIs restriction enzymes (Eschen-Lippold et al. 2014; Palm-Forster et al. 2012). The resulting sequences were shuttled into different destination vectors by Gateway^TM^-based cloning (Invitrogen): pE-SPYCE BiFC experiments in protoplasts; (Walter et al. 2004), pUBC-CFP/GFP localization studies in protoplasts and *N. benthamiana*; (Grefen et al. 2010), pUGW14 transient expression in protoplasts; (Nakagawa et al. 2007), pDEST22/pDEST32 (Y2H experiments, Invitrogen), pDEST-N110 recombinant protein expression in *E. coli*; (Dyson et al. 2004), pEARLEYGATE203 stable expression in plants; (Earley et al. 2006). For luciferase reporter assays, the promoter in the pFRK1-LUC plasmid (Asai et al. 2002) was exchanged against promoters of *PRP* (1.1 kb upstream of ATG), *PH1* (1.8 kb) and *PH2* (1.4 kb) by restriction enzyme-mediated cloning (*BamH*I and *Nco*I). All primers are listed in Supplemental Table 1.

### Yeast two-Hybrid experiments

The Gal4 reporter-based ProQuest^TM^ two-hybrid system (Invitrogen) was used to detect interactions of PRP, PH1 or PH2 wild type and MAPK docking site mutants with MAPKs. Assays were performed as described (Pecher et al. 2014).

### Transient expression in *Arabidopsis* protoplast and *N. benthamiana*

*Arabidopsis* mesophyll protoplasts were isolated and transformed according to reference (Yoo et al. 2007). Luciferase reporter assays were performed as described (Ranf et al. 2011; Sheikh et al. 2016) using protoplast samples co-expressing PRP/PH1/PH2 promoter-luciferase reporter constructs and pUBQ10-GUS for normalization (Sun and Callis 1997). For protein stability assays in protoplasts, pUGW14-PRP/PH1/PH2 or their phosphosite mutant/phospho-mimic versions were transformed. Samples were treated either with 100 nM flg22 alone or in combination with 1 µM cycloheximide to block protein translation and harvested at the indicated time points for subsequent Western blot analyses. For BiFC experiments in protoplasts, pE-SPYNE-MPK3/MPK4/MPK11 or pUC-SPYNE-MPK6 (Pecher et al. 2014) were co-transformed with pE-SPYCE-PRP/PH1/PH2 (wild type versions). To quantify interaction between MPK6 and PRP/PH1/PH2 wild type or MAPK docking site mutant versions, a third plasmid (cauliflower mosaic virus 35S promoter-CFP construct, p35S-CFP) was co-transformed. For subcellular localization of PRP, PH1 and PH2 in *N. benthamiana*, Agrobacterium-mediated delivery was applied. Agrobacterium GV3101 cells carrying the desired plasmids were resuspended in infiltration medium (10 mM MgCl_2_, 150 µM acetosyringone, 10 mM MES, pH 5.5), infiltrated into leaves (OD_600_ = 0.5) and 36–48 h later, the infiltrated area used for fluorescence microscopy.

### Microscopy

Microscopical analyses were conducted using an LSM710 confocal laser scanning system (Carl Zeiss; YFP excitation wavelength 514 nm and emission filter band-pass 520–540 nm; CFP excitation wavelength 458 nm and emission filter band-pass 465–500 nm; chloroplast auto-fluorescence band-pass 650–710 nm). To calculate interaction efficiencies in BiFC experiments, total fluorescence intensities of YFP (as a measure for interaction) and CFP (constitutive expression of free CFP for normalization) were measured for individual protoplasts. Values are given as relative signal intensities (YFP/CFP ratios).

### In vitro kinase assays with recombinant proteins

Preparation of recombinant proteins and in vitro kinase assay conditions using radioactive γ^32^P-ATP were described earlier (Feilner et al. 2005; Sheikh et al. 2016). To activate MPK3 and MPK6, a constitutively active variant of parsley MKK5 (PcMKK5-DD) was included (Lee et al. 2004).

### Western blot experiments

Protoplast samples were harvested by centrifugation and the cell pellets were directly frozen in liquid nitrogen. For immunoblot detection, pellets were mixed with standard SDS-loading dye, boiled at 95 °C for 5 min and subjected to SDS-PAGE (depending on the protein sizes 15 or 12% acrylamide gels were used). Proteins were blotted onto nitrocellulose membranes and proteins of interest were detected using α-HA.11 (clone 16B12; Covance) or α-cMyc (clone 9E10; Sigma Aldrich). Membranes were stained with amido black to assess equal loading.

### *Pseudomonas syringae* pv. *tomato* DC3000 infection experiments

For infection experiments, 3 week-old plants grown on soil were used. *Pseudomonas syringae* pv. *tomato* DC3000 was cultured over night at 28 °C in standard liquid Kings B medium. Cells were pelleted by centrifugation and subsequently resuspended in water to an OD_600_ = 1 (5 × 10^8^ CFU ml^−1^). Prior to spraying, 0.04% (v/v) Silwet-L77 was added. Leaf discs were harvested using a cork borer (0 and 3 dpi) and ground with a defined volume of water in a bead mill to release the bacteria. Serial dilutions were plated on LB agar with appropriate antibiotics. CFU were counted after incubation at 28 °C for 2 days.

### H_2_O_2_ (ROS) measurement and quantitative RT-PCR expression analysis

H_2_O_2_ accumulation after flg22 treatment of leaf discs and gene expression in seedlings were measured as previously described (Maldonado-Bonilla et al. 2014).

### Statistical analyses

All statistical tests were performed with GraphPad Prism 5 Software.

## Electronic supplementary material

Below is the link to the electronic supplementary material.

Supplementary material 1 (PDF 1773 KB)

## Abbreviations

PRP: Proline/serine-rich protein
PH1: PRP Homolog 1
PH2: PRP Homolog 2
MAPK or MPK: Mitogen-activated protein kinase
MPK3 or MPK6: Mitogen-activated protein kinase-3 or -6
MAMPs/PAMPs: Microbe- or pathogen-associated molecular patterns
BiFC: Bimolecular fluorescence complementation
Y2H: Yeast two-Hybrid
