# Supplementary material for: A novel family of proline/serine-rich proteins, which are phospho-targets of stress-related mitogen-activated protein kinases, differentially regulates growth and pathogen defense in Arabidopsis thaliana
Source: Plant Mol Biol. 2017 Jul 28;95(1):123–40. doi: 10.1007/s11103-017-0641-5 (PMC5594048; doi:10.1007/s11103-017-0641-5)
Supplement: Supplementary file 1 — Supplementary material 1 (PDF 1773 KB) [file 11103_2017_641_MOESM1_ESM.pdf]

Supplemental Figure 1

Fig. S1

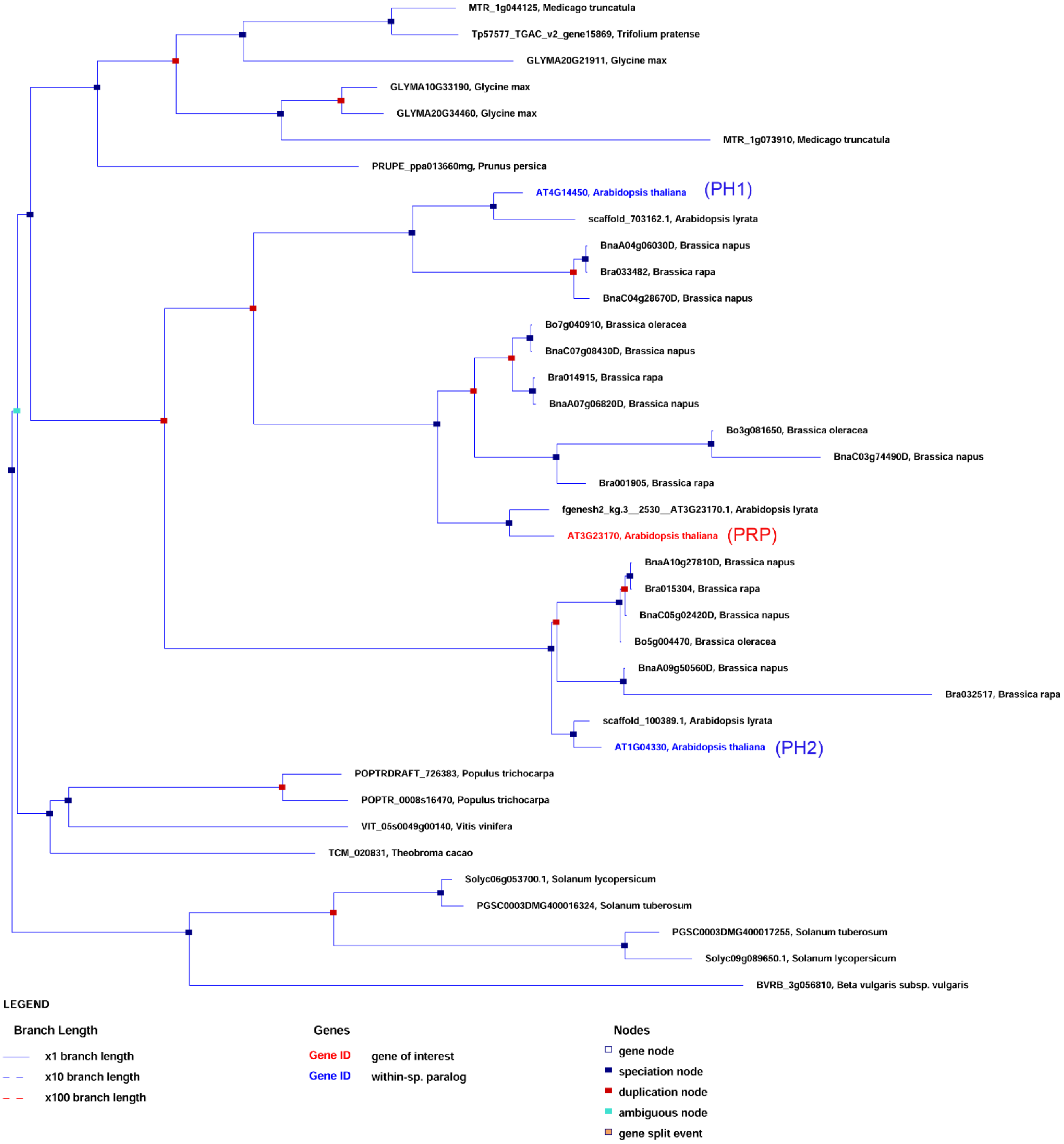

Fig. S1: Phylogenetic tree created by EnsemblPlants (http://plants.ensembl.org/Arabidopsis_thaliana/Info/Index).
PRP, its two paralogs and the putative PRP orthologues are labelled in red, blue or black fonts, respectively.

Supplemental Figure 2

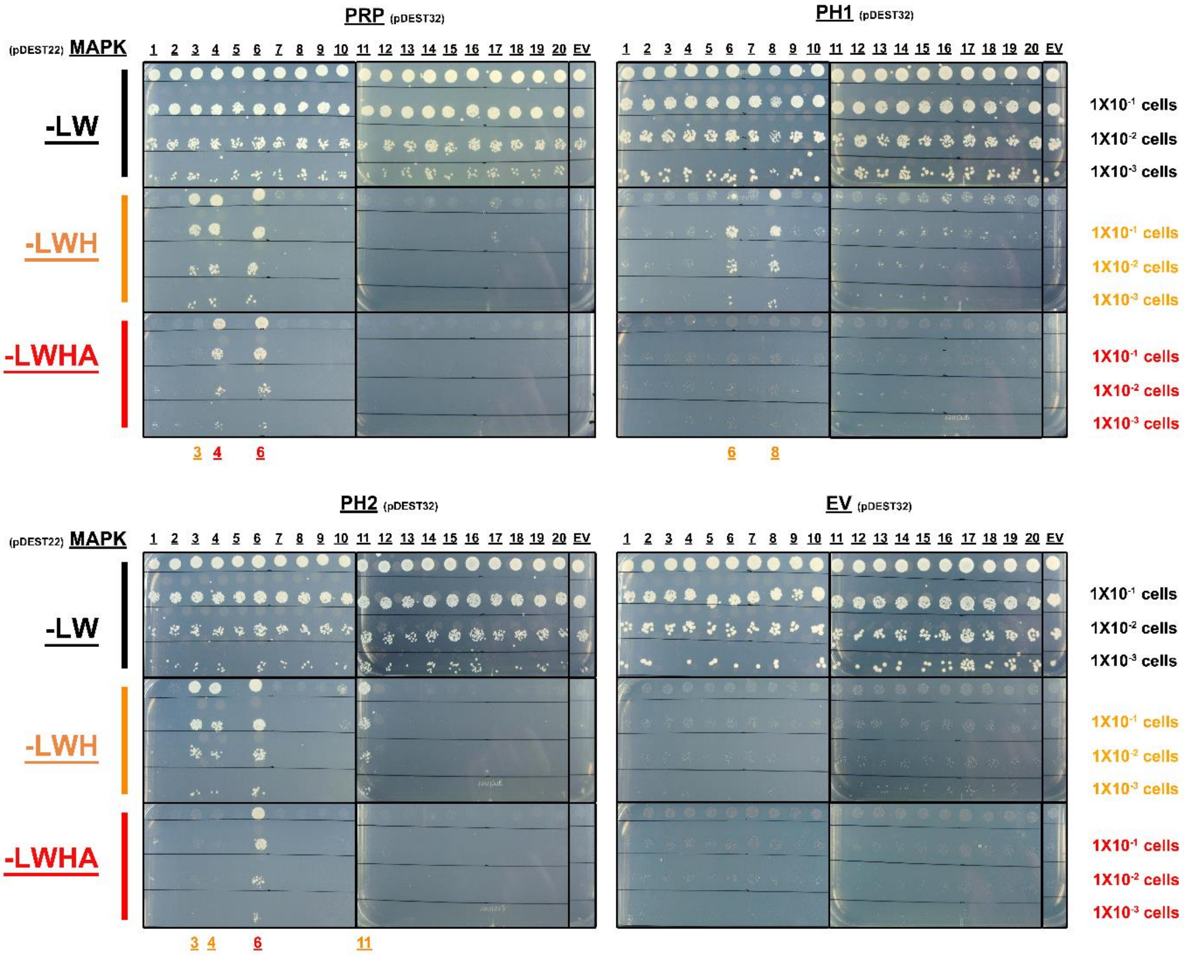

Fig. S2

Y2H screen of PRP, PH1 and PH2 against all 20 Arabidopsis MAPKs (performed as described in Fig. 2a). Positive interactions are indicated by yeast growth on selective synthetic drop-out media (SD -Leu/-Trp/-His [-LWH] or -Leu/-Trp/-His/-Ade [-LWHA]); EV= empty vector control. Note that figure was assembled from different plates.

Supplemental Figure 3

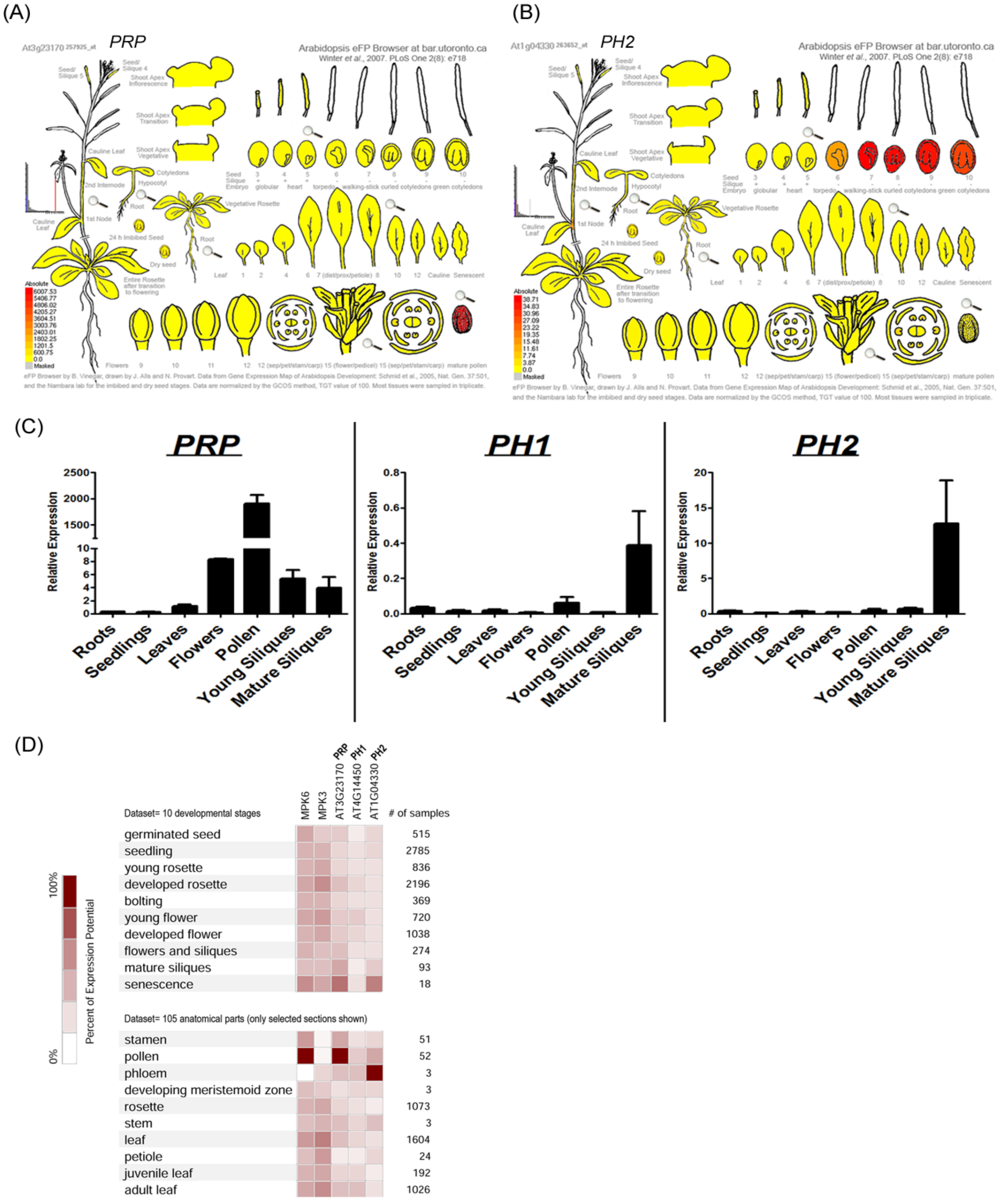

Fig. S3 Anatomical expression profile of PRP, PH1 and PH2.
Anatomical expression profile of PRP, PH1 and PH2. **A, B**: eFP-Browser (http://bar.utoronto.ca/efp/cgi-bin/efpWeb.cgi) pictograms denoting *PRP* and *PH1* levels in various organs and tissues (Note that no information is available for *PH2*). **C**. Quantitative RT-PCR expression studies of transcript levels in the indicated tissues. **D.** Comparison of *MPK3/6*, *PRP*, *PH1* and *PH2* expression potential in the indicated tissue types (as extracted from all the microarray experiments available in the Genevestigator platform). The number of microarray samples used for the prediction is shown on the right.

Supplemental Figure S4

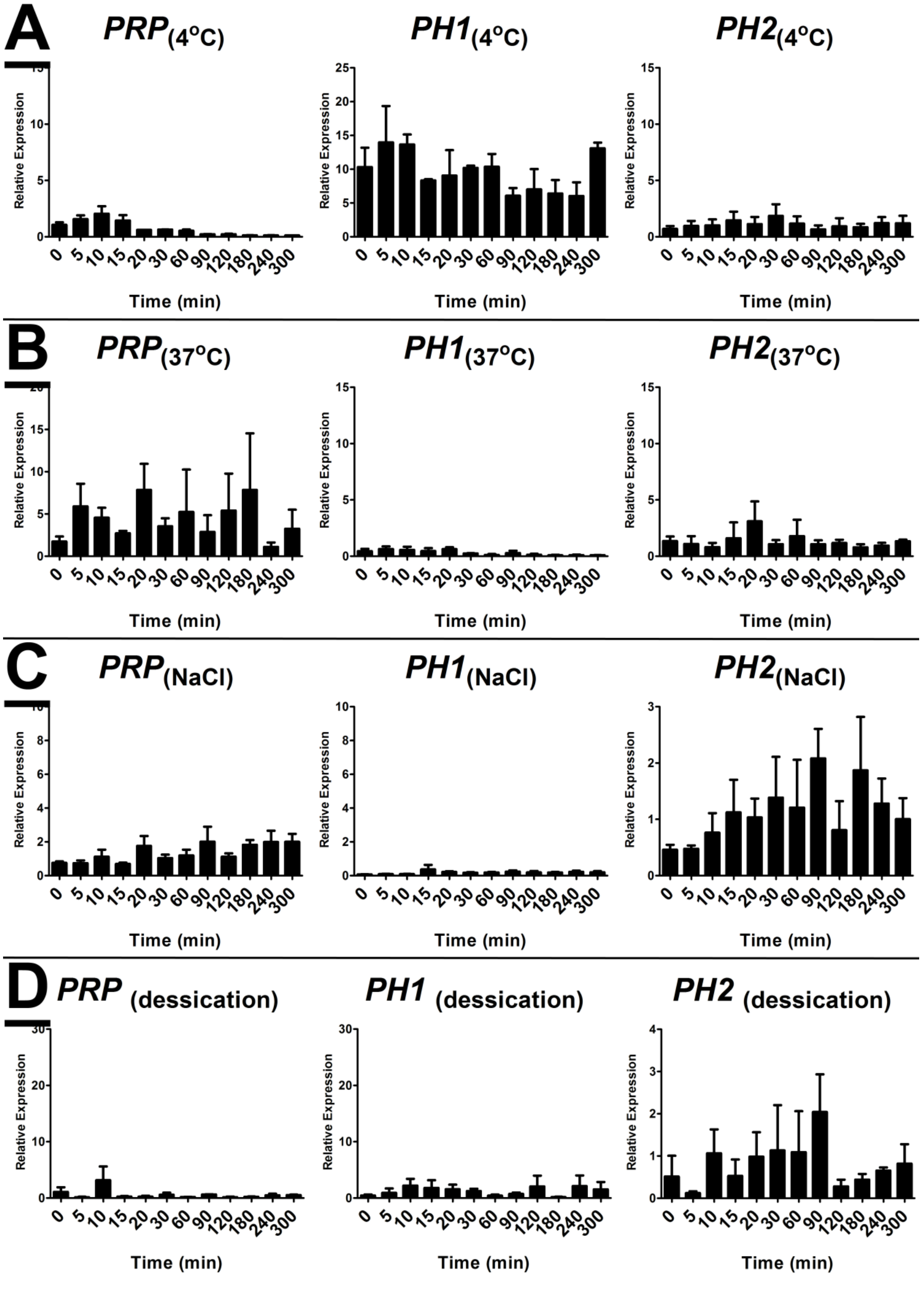

Fig. S4 Expression analyses of PRP, PH1 and PH2 in seedlings upon abiotic stress treatments.

Quantitative RT-PCR expression analyses of *PRP*, *PH1* and *PH2* in seedlings upon various abiotic stress treatments. Seedlings were grown in liquid culture for two weeks in 24-well microtiter plates. **A.** Cold treatment (4°C) was performed by exchanging the medium against fresh precooled medium and placing the plate on ice. **B.** Heat stress (37°C) was induced by exchanging the medium against preheated medium and incubating the plate in an adjusted incubator (37°C). **C.** Salt stress was induced by adding NaCl to a final concentration of 250 mM. **D.** Drought stress (desiccation) was induced by removing seedlings from the media and placing them on paper tissue to absorb excess liquid, forcing them to dry during the time course of the experiment. Samples were harvested at the indicated time points. Total RNA was isolated, reverse transcribed and used for quantitative realtime PCR to measure *PRP*, *PH1* and *PH2* transcript levels relative to PP2A. No statistical significant differences were observed between the samples (n = 4; Kruskal-Wallis One-way ANOVA with Dunns posttest). The experiment was repeated twice with similar results.

Supplemental Figure S5

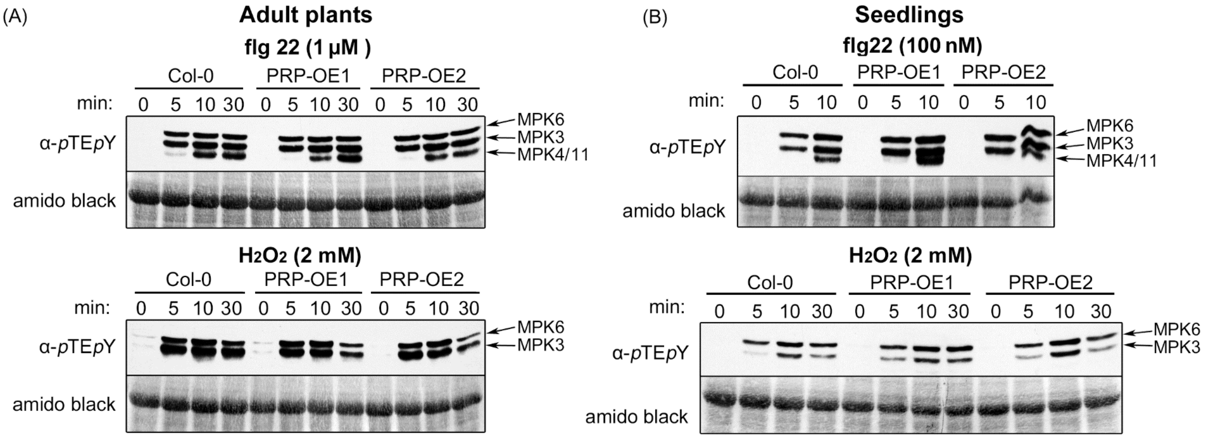

Fig. S5 MAPK activation profile in PRP-OE lines compared to Col-0 wild-type plants.
MAPK activation profile was visualized by immunoblot with antibodies recognizing the phosphorylated "TEY" motif within the activation loop. Three-week-old adult plants (**A**) or 10 day-old seedlings (**B**) were treated with flg22 or H_2O_2 and collected at the indicated timepoints. Amido black staining of the Rubisco large subunit band is used to gauge equal loading.

Supplementary Table 1 Oligonucleotide primers used in the study.

name	sequence	purpose
PRP_5	5'-CACCATGTCGACGACGATGAAGAG-3'	cloning into pENTR™/D-TOPO®
PRP_stop_3	5'-CTAAACCGGAACAAACGGTG-3'	cloning into pENTR™/D-TOPO®
PRP_nostop_3	5'-AACCGGAACAAACGGTG-3'	cloning into pENTR™/D-TOPO®
PH1_5	5'-CACCATGTTCTTTGATACAAAAGTACTCAA-3'	cloning into pENTR™/D-TOPO®
PH1_stop_3	5'-CTAAACCTGGATGAACGGTG-3'	cloning into pENTR™/D-TOPO®
PH1_nostop_3	5'-AACCTGGATGAACGGTG-3'	cloning into pENTR™/D-TOPO®
PH2_5	5'-CACCATGTCGTCCACGGCGAGA-3'	cloning into pENTR™/D-TOPO®
PH2_stop_3	5'-TTAATTTGGCCATGCAAACG-3'	cloning into pENTR™/D-TOPO®
PH2_nostop_3	5'-ATTTGGCCATGCAAACG-3'	cloning into pENTR™/D-TOPO®
PRP_qPCR_5	5'-GTGGCGGTTGAGAAAAGA-3'	quant. realtime PCR; SYBRgreen
PRP_qPCR_3	5'-GAACAAACGTCGATGACTCG-3'	quant. realtime PCR; SYBRgreen
PH1_qPCR_5	5'-GGTGAAGAAGACACCAGTTTTCA-3'	quant. realtime PCR; SYBRgreen
PH1_qPCR_3	5'-GTTGTTGGCTCGTAGCAGAA-3'	quant. realtime PCR; SYBRgreen
PH2_qPCR_5	5'-GTCGCCACCACAAAAACC-3'	quant. realtime PCR; SYBRgreen
PH2_qPCR_3	5'-GCTTCTCCGCCTCTTTACC-3'	quant. realtime PCR; SYBRgreen
PP2A_qPCR_5	5'-GACCGGAGCCAACTAGGAC-3'	quant. realtime PCR; SYBRgreen
PP2A_qPCR_3	5'-AAAACTTGGTAACTTTTCCAGCA-3'	quant. realtime PCR; SYBRgreen
NHL10_qPCR_5	5'-ACGCCGGACAGTCTAGGA-3‘	quant. realtime PCR; SYBRgreen
NHL10_qPCR_3	5’-CCCTAAGCCTGAACTTGATCTC-3‘	quant. realtime PCR; SYBRgreen
PAD3_qPCR_5	5’-CACCACTGATCATCTCAAAGGA-3‘	quant. realtime PCR; SYBRgreen
PAD3_qPCR_3	5’-CGGTCATTCCCCATAGTGTT-3’	quant. realtime PCR; SYBRgreen
ZAT12_qPCR_5	5’-CTTTGGGAGGACACATGAGG-3‘	quant. realtime PCR; probe-based
ZAT12_qPCR_3	5’-CAAAGCGCGTGTAACCAAC-3’	quant. realtime PCR; probe-based
ZAT12_probe	Roche Universal ProbeLibrary probe: #66, cat.no. 04688651001	quant. realtime PCR; probe-based
PRP_promotor_5	5'-GGATCCTCATCGCTTCTTTTCTTCTTTGT-3'	cloning of PRP promoter
PRP_promotor_3	5'-CCATGGAAACGCGAAAGAAATCGAGTT-3'	cloning of PRP promoter
PH1_promotor_5	5'-GGATCCGGTGCGTACGTTAAGGACTTACT-3'	cloning of PH1 promoter
PH1_promotor_3	5'-CCATGGAATAATGATGTTGGTGTGTGATGT-3'	cloning of PH1 promoter
PH2_promotor_5	5'-GGATCCAGTTCTTCTCTTCTCCTCCACACA-3'	cloning of PH2 promoter
PH2_promotor_3	5'-CCATGGAGCAGGATCTTGTTGTATTAATTT-3'	cloning of PH2 promoter
PRP_S51D_5	5'-atatGCTCTTCCGACCCGTTAGCTCTTTCTCCC-3'	generation of PRP-S51D
PRP_S51D_3	5'-ttttGCTCTTCGGTCGAGGAGAGGTATAGCCGT-3'	generation of PRP-S51D
PH1_S65D_5	5'-atatGCTCTTCTGACCCGCTCGCTCCTTCTCTA-3'	generation of PH1-S65D
PH1_S65D_3	5'-ttttGCTCTTCGGTCAAGGAGAGGGATCGCCAC-3'	generation of PH1-S65D
PH2_S44D_5	5'-atatGCTCTTCAGACCCTACGGAGTCGCCACCA-3'	generation of PH2-S44D
PH2_S44D_3	5'-ttttGCTCTTCGGTCTAGAAGAGGAATAGCCAC-3'	generation of PH2-S44D
PRPdock_F	5'-AAGGTCTCCCCCCGGCGGATAAAGACGTTC-3‘	generation of PRP-DSM
PRPdock_R	5'-AAGGTCTCCGGGGGTTCTTCTAGTAGACGA-3‘	generation of PRP-DSM
PH1dock_F	5'-AAGGTCTCCTCCGTCTGATATGGACAAACC-3‘	generation of PH1-DSM
PH1dock_R	5'-AAGGTCTCCGGAGCTTCTTCCTGTAGCTGGCTAAGTTGCC-3‘	generation of PH1-DSM
PH2dock_F	5'-AAGGTCTCCTCCACCGCCTGATAAGGAAAACCCTTGCGAAGCG-3‘	generation of PH2-DSM
PH2dock_R	5'-AAGGTCTCTGGAGCTTCTTCCTGCAGCCTA-3‘	generation of PH2-DSM

Footnotes:
CACC= 5’-extension for directional cloning into pENTR™/D-TOPO® vector (Invitrogen)
GGATCC / CCATGG= *BamH*I / *Nco*I restriction sites for directional cloning
GCTCTTC / GGTCTC= *Lgu*I / *Eco*31I type IIs restriction sites
FAM= 6-carboxyfluorescein fluorescent dye
Cy®5= far-red-fluorescent dye
BHQ1 / BBQ= Black Hole Quencher 1® / BlackBerry® Quencher
DSM= MAPK docking site mutant

Note that primers used to generate phosphosite mutants were already described in Palm-Forster et al. (2012).

Reference

Palm-Forster MAT, Eschen-Lippold L, Lee J (2012) A mutagenesis-based screen to rapidly identify phosphorylation sites in mitogen-activated protein kinase substrates Anal Biochem 427:127-129 doi: 10.1016/J.Ab.2012.05.015
